# CmHY5-CmWRKY23/69-*CmGH9B3* module mediates red light promoted graft union healing of melon grafted onto squash

**DOI:** 10.1093/hr/uhaf251

**Published:** 2025-09-17

**Authors:** Yulei Zhu, Jianyu Li, Hongxi Wu, Jiahui Wang, Heng Wang, Chuanqiang Xu, Tianlai Li

**Affiliations:** College of Horticulture, Shenyang Agricultural University, Shenyang 110866, China; Key Laboratory of Protected Horticulture of Education of Ministry and Liaoning Province, National & Local Joint Engineering Research Center of Northern Horticultural Facilities Design & Application Technology, Shenyang 110866, China; Key Laboratory of Horticultural Equipment, Ministry of Agriculture and Rural Affairs, Shenyang 110866, China; Modern Protected Horticulture Engineering & Technology Center, Shenyang 110866, China; Stockbridge School of Agriculture, University of Massachusetts Amherst, Amherst, MA 01003, USA; College of Horticulture, Shenyang Agricultural University, Shenyang 110866, China; Key Laboratory of Protected Horticulture of Education of Ministry and Liaoning Province, National & Local Joint Engineering Research Center of Northern Horticultural Facilities Design & Application Technology, Shenyang 110866, China; Key Laboratory of Horticultural Equipment, Ministry of Agriculture and Rural Affairs, Shenyang 110866, China; Modern Protected Horticulture Engineering & Technology Center, Shenyang 110866, China; College of Horticulture, Shenyang Agricultural University, Shenyang 110866, China; Key Laboratory of Protected Horticulture of Education of Ministry and Liaoning Province, National & Local Joint Engineering Research Center of Northern Horticultural Facilities Design & Application Technology, Shenyang 110866, China; Key Laboratory of Horticultural Equipment, Ministry of Agriculture and Rural Affairs, Shenyang 110866, China; Modern Protected Horticulture Engineering & Technology Center, Shenyang 110866, China; College of Horticulture, Shenyang Agricultural University, Shenyang 110866, China; Key Laboratory of Protected Horticulture of Education of Ministry and Liaoning Province, National & Local Joint Engineering Research Center of Northern Horticultural Facilities Design & Application Technology, Shenyang 110866, China; Key Laboratory of Horticultural Equipment, Ministry of Agriculture and Rural Affairs, Shenyang 110866, China; Modern Protected Horticulture Engineering & Technology Center, Shenyang 110866, China; College of Horticulture, Shenyang Agricultural University, Shenyang 110866, China; Key Laboratory of Protected Horticulture of Education of Ministry and Liaoning Province, National & Local Joint Engineering Research Center of Northern Horticultural Facilities Design & Application Technology, Shenyang 110866, China; Key Laboratory of Horticultural Equipment, Ministry of Agriculture and Rural Affairs, Shenyang 110866, China; Modern Protected Horticulture Engineering & Technology Center, Shenyang 110866, China; College of Horticulture, Shenyang Agricultural University, Shenyang 110866, China; Key Laboratory of Protected Horticulture of Education of Ministry and Liaoning Province, National & Local Joint Engineering Research Center of Northern Horticultural Facilities Design & Application Technology, Shenyang 110866, China; Key Laboratory of Horticultural Equipment, Ministry of Agriculture and Rural Affairs, Shenyang 110866, China; Modern Protected Horticulture Engineering & Technology Center, Shenyang 110866, China

## Abstract

Grafting is extensively utilized in melon (*Cucumis melo* L.) cultivation to improve environmental tolerance and disease resistance. Our previous studies identified *CmGH9B3*, encoding β-1,4-glucanase, as a key factor promoting cell adhesion during graft union formation in melon scions grafted onto squash rootstocks. However, the upstream regulatory mechanisms controlling *CmGH9B3* expression remained unclear. Here, we demonstrate that LED red light significantly enhances graft union healing by activating a transcriptional module involving CmHY5, CmWRKY23, CmWRKY69, and *CmGH9B3*. Specifically, the light-responsive transcription factor CmHY5 was induced by LED red light and activated the expression of CmWRKY23 and CmWRKY69. These WRKY transcription factors were directly bound to the *CmGH9B3* promoter, promoting its expression to accelerate vascular reconnection and graft healing. Our findings establish a mechanistic link between light signaling and graft union formation via the CmHY5-CmWRKY23/69-*CmGH9B3* regulatory module, offering practical targets to improve grafting efficiency in melon cultivation.

## Introduction

Melon (*Cucumis melo* L.) serves as a valuable annual horticultural crop within the Cucurbitaceae family [1]. However, melon production also faces several challenges, including soil-borne diseases and the poor cold resistance of the root system [2]. Grafting has become the primary method of melon production, creating considerable economic value. The success of this practice depends largely on graft union formation, particularly tissue adhesion and healing, which are critical for seedling survival and overall graft performance [3]. Recent research has emphasized elucidating the molecular mechanisms of graft union healing and identifying pivotal regulatory elements. Within these elements, β-1,4-glucanase enzymes are crucial for altering plant cell walls and facilitating intercellular adhesion [4]. In melon, our previous work showed that expression of *CmGH9B3*, which encodes a β-1,4-glucanase, was up-regulated under supplemental light and associated with improved healing in melon scions grafted onto squash rootstocks [5]. Homologous GH9B3 genes have also been shown to enhance graft compatibility in tobacco, soybean, and petunia, and to facilitate xylem bridge formation in parasitic plants like *Schistosoma japonicum* [6, 7].

The microclimate, especially light conditions, is crucial in controlling the formation of graft unions in grafted seedlings [8]. Light is essential in determining plant development by influencing processes such as seed germination, de-etiolation, hormone distribution, and organ growth [9]. It modulates hormonal pathways, particularly auxin transport, by activating photoreceptor-dependent signaling cascades [10, 11]. These light signals induce the expression of numerous transcription factor families, including bZIP, bHLH, MYB, GATA, GT1, and others [12, 13]. HY5, a crucial light-responsive protein, is a bZIP protein that accumulates in reaction to visible and UV-B light. It plays a key role in promoting photomorphogenesis by activating downstream target genes [14–16].

**Figure 1:**
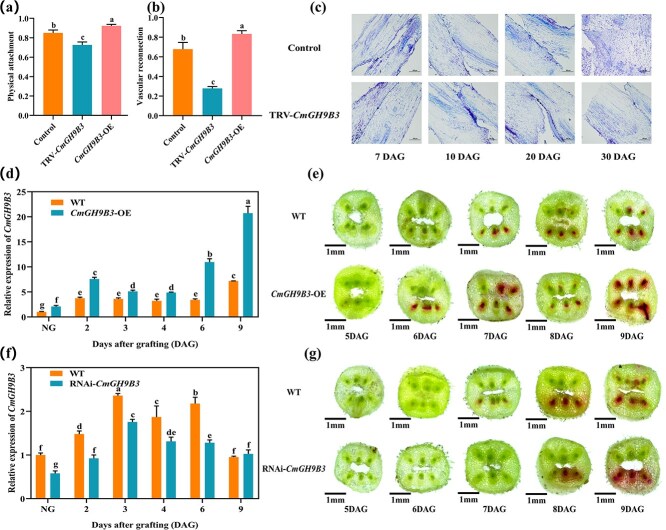
*CmGH9B3* promotes graft union healing in oriental melon scions grafted onto squash rootstocks. (a) Physical adhesion rate at 2 DAG in WT, *CmGH9B3*-overexpressing (OE), and TRV-*CmGH9B3* silenced seedlings. (b) Vascular connectivity rate at 6 DAG in the same lines. (c) Tissue sections showing graft interface structure in TRV-*CmGH9B3* and control plants at 4 DAG. Scale bar = 500 μm. (d) Relative expression of *CmGH9B3* in OE and WT plants during graft healing. (e) Acid fuchsin staining of xylem reconnection from 5 to 9 DAG in OE and WT plants. Scale bar = 1 mm. (f) Relative expression of *CmGH9B3* in RNAi lines compared to WT during graft healing. (g) Acid fuchsin staining of vascular connectivity in RNAi and WT plants at 6 DAG. Scale bar = 1 mm. Data represent mean ± SD (*n* = 3). Different letters indicate significant differences (ANOVA, *P* < 0.05).

Transcriptional regulation plays a critical role in enabling plants to react to environmental cues. WRKY transcription factors, identified by their conserved domain, serve as pivotal controllers in plant defense, hormone signaling, and development [17]. The proteins interact with W-box elements (TTGACC/T) located in promoter regions to modulate gene expression [18]. Early research has shown that WRKY proteins are crucial regulators in a range of developmental and physiological processes, such as leaf development, root growth, seed development, senescence, and the plant's reactions to biotic and abiotic stresses. For example, overexpression of WRKY12 in *Arabidopsis* or grape callus enhances antioxidant gene expression under cold stress [19], while other WRKYs modulate ABA-dependent signaling pathways in stress responses [20]. However, the function of WRKY transcription factors in the graft union healing process has not been established.

In this study, we investigated how LED red light regulates graft union healing in melon scions grafted onto squash rootstocks. Building upon previous transcriptomic findings, we posited that the β-1,4-glucanase gene *CmGH9B3* plays a pivotal role in this process and is subject to transcriptional regulation by upstream light-responsive factors. Our objectives were to (i) functionally characterize *CmGH9B3* during graft union healing, (ii) identify and validate its transcriptional regulators, and (iii) investigate whether red light influences this regulatory pathway through the light-signaling factor CmHY5.

## Results

### *CmGH9B3* promotes graft union healing in oriental melon scions grafted onto squash rootstocks

Our previous transcriptome analysis initially identified *CmGH9B3* as a candidate gene expressed during graft union healing in oriental melon scions grafted onto squash rootstocks [21]. To investigate its function, we used virus-induced gene silencing (TRV-*CmGH9B3*) in melon scions. Compared to controls, silenced plants showed reduced physical adhesion and vascular connectivity (Fig. 1a and b; Fig. S1a and b). In addition, we observed that the cell wall thickness of scion and rootstock cells at their grafting interface was significantly thicker (Fig. S1c), and toluidine blue staining revealed that cell reintegration was delayed in the silenced lines (Fig. 1c).

Transient lines (*CmGH9B3*-OE) were generated and confirmed by RT-qPCR (Quantitative Real-time PCR), which showed significantly elevated *CmGH9B3* expression compared to wild-type (WT) plants during the healing process (Fig. 1d; Fig. S1d). These OE plants also exhibited increased physical adhesion and vascular connectivity at early time points (Fig. 1a and b). To evaluate vascular reconnection, we performed acid fuchsin staining at 5, 6, 7, 8, and 9 days after grafting (DAG). In *CmGH9B3*-OE plants, xylem staining was first observed at 5 DAG and became progressively more extensive, with all xylem showing uptake by 8 DAG. In contrast, WT plants showed delayed staining, with only two xylems stained at 6 DAG and partial uptake persisting until 9 DAG (Fig. 1e).

Stable RNAi transgenic lines targeting *CmGH9B3* were generated and confirmed by kanamycin resistance, PCR, and sequencing (Fig. S1e and f). RT-qPCR analysis showed that *CmGH9B3* transcript levels were significantly reduced in multiple independent T1 lines (Fig. S1g and h). These RNAi lines grew normally but exhibited decreased *CmGH9B3* expression following grafting (Fig. 1f), and acid fuchsin staining exhibited a delay in xylem reconnection compared to WT plants (Fig. 1g).

### LED red light induces *CmGH9B3* expression and promotes graft union healing in oriental melon

**Figure 2:**
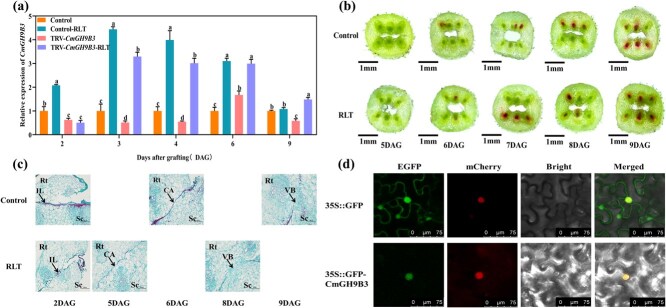
Red light treatment enhances *CmGH9B3* expression and graft healing. (a) Relative *CmGH9B3* expression in TRV-*CmGH9B3* seedlings under red light treatment vs. dark conditions at 2, 4, and 6 DAG. (b) Acid fuchsin staining showing enhanced vascular reconnection in red-light-treated plants. Scale bar = 1 mm. (c) Paraffin sections of graft interfaces at 6 DAG under dark and red light treatment. IL, isolation layer; CA, callus; VB, vascular bundle; Sc, scion; Rt, rootstock. Scale bar = 200 μm. (d) Subcellular localization of *CmGH9B3*-GFP fusion protein in *N. benthamiana* epidermal cells. GFP signal was nuclear localized; control GFP was cytoplasmic and nuclear. mCherry marks nuclear localization. Scale bar = 75 μm. RLT, Red light treatment. Different letters indicate significant differences (ANOVA, *P* < 0.05). Data are shown as mean ± SD (*n* = 3).

Our previous studies indicated that a light-responsive element was identified after the prediction of multiple cis-regulatory elements on the *CmGH9B3* promoter by PlantCARE [5]. TRV-*CmGH9B3* scions exposed to LED red light showed significantly higher *CmGH9B3* expression compared to the dark control, partially restoring expression of silenced lines (Fig. 2a). *CmGH9B3* expression was highest in seedlings treated with 90 min of daily red light, which also resulted in the least visible scion wilting. This treatment condition was used in subsequent experiments (Fig. S2). Acid fuchsin staining revealed increased vascular connectivity in red-light-treated grafted seedlings compared to controls (Fig. 2b). Histological sections of the graft interface showed earlier degradation of the isolation layer, enhanced callus formation, and more continuous development of vascular bundles under red light (Fig. 2c). The subcellular localization of the *CmGH9B3* protein was determined by transiently expressing a *CmGH9B3*-GFP fusion in *Nicotiana benthamiana*. The GFP signal was observed solely in the nucleus, in contrast to the control GFP, which was dispersed throughout the cell, supporting the nuclear localization of *CmGH9B3* (Fig. 2d).

### CmWRKY23 and CmWRKY69 bind the *CmGH9B3* promoter and activate its expression

**Figure 3:**
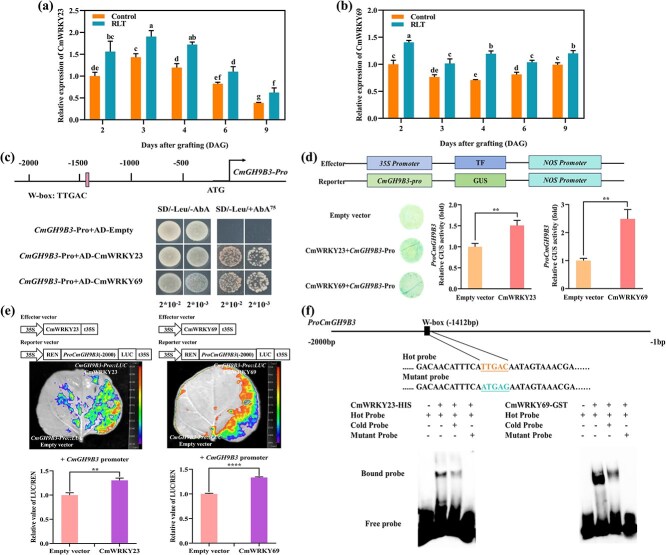
Transcription factors CmWRKY23 and CmWRKY69 directly bind and activate the *CmGH9B3* promoter. (a, b) Expression of CmWRKY23 and CmWRKY69 in melon stems under red light treatment vs. dark at 2, 4, and 6 DAG. (c) Y1H assay showing binding of CmWRKY23 and CmWRKY69 to the *CmGH9B3* promoter. (d) GUS reporter assay in *N. benthamiana* co-infiltrated with *CmGH9B3* promoter::GUS and CmWRKY effectors. (e) Luciferase reporter assay showing activation of *CmGH9B3* promoter by CmWRKY23 and CmWRKY69 *in vivo*. (f) EMSA demonstrating specific binding of CmWRKY23-HIS and CmWRKY69-GST to W-box elements in the *CmGH9B3* promoter. RLT, Red right treatment. Error bars represent mean ± SD (*n* = 3). Statistical significance: ^**^P < 0.01 (*t*-test).

Transcriptome analysis of healing graft junctions revealed that CmWRKY23 and CmWRKY69 were among the most highly expressed WRKY family members during the early stages of graft union development. RT-qPCR confirmed that both transcripts were significantly higher in red-light-treated seedlings compared to dark controls (Fig. 3a and b).

Following the observation that *CmGH9B3* transcript levels increased under red light, we analyzed its promoter region to identify potential upstream regulatory elements. Within the 2.0-kb upstream sequence, multiple W-box motifs (TTGAC), known binding sites for WRKY transcription factors, were identified. Yeast one-hybrid (Y1H) assays demonstrated that CmWRKY23 and CmWRKY69 each bind directly to the *CmGH9B3* promoter (Fig. 3c). GUS reporter assays in *N. benthamiana* leaves further supported this interaction: co-expression of CmWRKY23 or CmWRKY69 with a *CmGH9B3* promoter::GUS construct led to significantly increased GUS activity compared to the control (Fig. 3d). Luciferase reporter assays confirmed transcriptional activation *in vivo*. Co-expression of CmWRKY23 or CmWRKY69 with the *CmGH9B3* promoter::LUC construct significantly enhanced luminescence relative to controls (Fig. 3e).

Electrophoretic mobility shift assays (EMSA) showed that CmWRKY23-HIS and CmWRKY69-GST fusion proteins bound specifically to W-box-containing probes from the *CmGH9B3* promoter. Binding was reduced by competition with unlabeled probes and abolished with mutated sequences, confirming specificity (Fig. 3f).

### CmWRKY23 and CmWRKY69 promote graft healing by activating *CmGH9B3*

**Figure 4:**
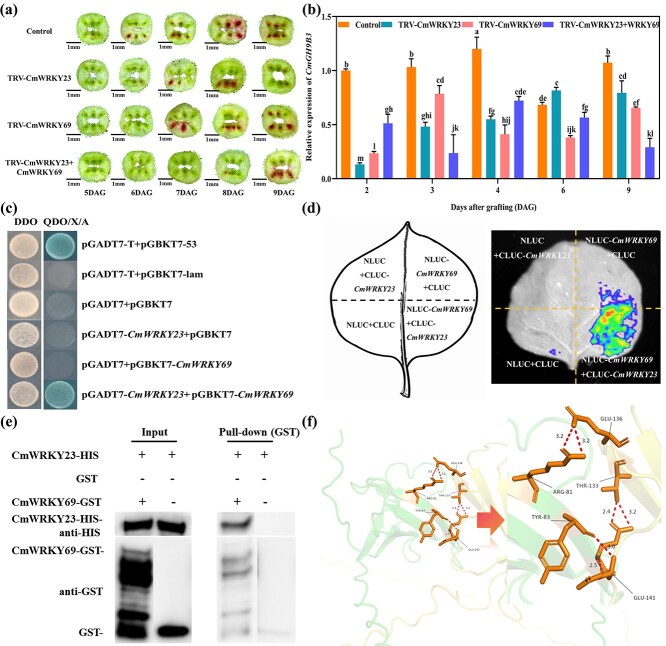
CmWRKY23 and CmWRKY69 functionally interact and regulate *CmGH9B3*. (a) Acid fuchsin staining showing delayed vascular reconnection in TRV-CmWRKY23, TRV-CmWRKY69, and dual-silenced plants at 6 DAG. Scale bar = 1 mm. (b) Relative *CmGH9B3* expression in silenced plants compared to control. (c) Y2H assay demonstrating interaction between CmWRKY23 and CmWRKY69. (d) LCI assay confirming CmWRKY23–CmWRKY69 interaction in *N. benthamiana*. (e) Pull-down assay showing *in vitro* interaction between CmWRKY23-HIS and CmWRKY69-GST. (f) AlphaFold3-predicted structure of the CmWRKY23–CmWRKY69 complex bound to DNA, highlighting seven predicted contact residues. Error bars represent mean ± SD (*n* = 3). Different letters indicate significant differences (ANOVA, *P* < 0.05).

To assess the role of CmWRKY23 and CmWRKY69 in graft healing, we transiently silenced each gene in melon scions using virus-induced gene silencing (TRV-CmWRKY23, TRV-CmWRKY69) either individually or in combination. RT-qPCR confirmed reduced transcript levels of CmWRKY23 and CmWRKY69 in the respective silenced lines (Fig. S3a–d). The expression of *CmGH9B3* was also reduced in these silenced plants, which is consistent with the role of these WRKY transcription factors in regulating its expression (Fig. 4b; Fig. S3e–g). Acid fuchsin staining showed delayed xylem reconnection in TRV-CmWRKY23, TRV-CmWRKY69, and TRV-CmWRKY23 *+* CmWRKY69 plants compared to the control (Fig. 4a), indicating impaired vascular connectivity. To determine whether CmWRKY23 and CmWRKY69 physically interact, we performed a yeast two-hybrid (Y2H) assay. CmWRKY23 and CmWRKY69 proteins interacted in yeast (Fig. 4c), and this interaction was further confirmed *in planta* using luciferase complementation imaging (LCI) in *N. benthamiana* (Fig. 4d). A pull-down assay using recombinant CmWRKY23-HIS and CmWRKY69-GST fusion proteins also verified their interaction *in vitro* (Fig. 4e).

We modeled the CmWRKY23-CmWRKY69 protein complex using AlphaFold3 to identify putative interaction sites. The predicted structure revealed seven amino acid residues in CmWRKY23 involved in protein–protein docking with CmWRKY69 within a 4 Å interface (Fig. 4f).

### CmHY5 activates CmWRKY23 and CmWRKY69 transcription through promoter binding

**Figure 5:**
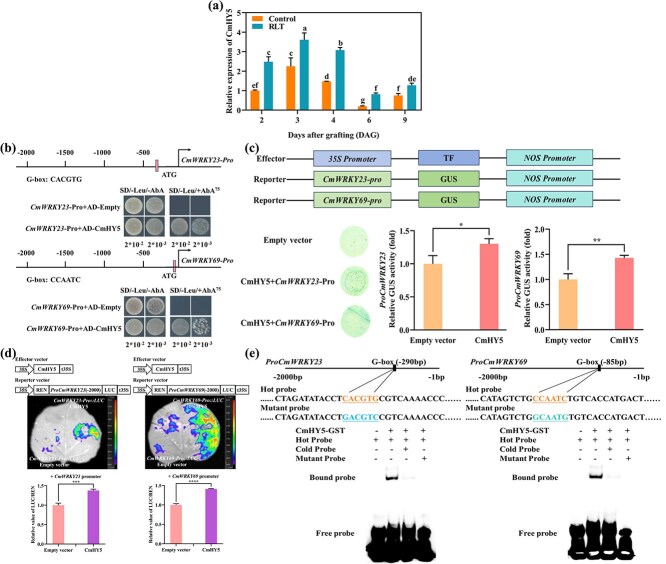
CmHY5 binds the promoters of CmWRKY23 and CmWRKY69 and activates their expression. (a) Relative expression of CmHY5 at 2, 4, and 6 DAG under red light treatment vs. dark conditions. (b) Y1H assay showing CmHY5 binding to CmWRKY23 and CmWRKY69 promoters. (c) GUS activity assay in *N. benthamiana* leaves co-infiltrated with HY5 and WRKY promoter constructs. (d) Luciferase assay confirming HY5-mediated activation of CmWRKY23 and CmWRKY69 promoters *in vivo*. (e) EMSA showing CmHY5 binding to G-box motifs in CmWRKY23 and CmWRKY69 promoters. Binding was reduced with unlabeled competitors and abolished with mutated probes. RLT, Red right treatment. Data are mean ± SD (*n* = 3). ^**^P < 0.01 (*t*-test).

To determine whether CmHY5 regulates the transcription of CmWRKY23 and CmWRKY69, we examined their expression in red-light-treated grafted seedlings. RT-qPCR showed that CmHY5 transcript levels were significantly higher in scions exposed to red light compared to dark controls (Fig. 5a). Y1H assays revealed that CmHY5 binds directly to the promoters of CmWRKY23 and CmWRKY69 (Fig. 5b). GUS reporter assays in *N. benthamiana* showed significantly increased activity when CmHY5 was co-expressed with either CmWRKY23 or CmWRKY69 promoter::GUS constructs (Fig. 5c). Luciferase reporter assays confirmed these interactions *in vivo*: co-infiltration of CmHY5 with CmWRKY23 or CmWRKY69 promoter::LUC constructs enhanced luminescence relative to controls (Fig. 5d).

EMSA further demonstrated that CmHY5 binds specifically to G-box-like motifs (CACGTG and CCAATC) in the promoters of CmWRKY23 and CmWRKY69. Binding was reduced by excess unlabeled probe and abolished by mutant probes, confirming binding specificity (Fig. 5e).

### CmHY5 promotes graft union healing by activating *CmWRKY23, CmWRKY69,* and downstream *CmGH9B3*

**Figure 6:**
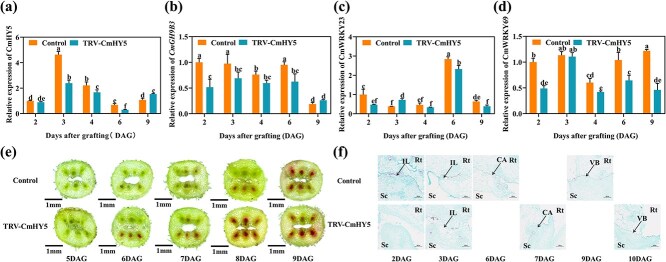
CmHY5 promotes graft healing via CmWRKY23, CmWRKY69, and *CmGH9B3*. (a–d) Relative expression of *CmGH9B3*, CmWRKY23, and CmWRKY69 in oriental melon stems after transient silencing of CmHY5. Different letters indicate significant differences (*P* < 0.05). Values are means ± SD, *n* = 3. (e) Observation of acid fuchsin absorption during the graft union healing of oriental melon scion grafted onto squash rootstock (TRV-CmHY5, transient silent CmHY5 conditions; scale bars, 1 mm). (f) Paraffin section observation during the graft union healing of oriental melon scion grafted onto squash rootstock (IL, the isolated layer; CA, the callus; VB, the vascular bundles; Sc, scion; Rt, rootstock; TRV-CmHY5, transient silent CmHY5 conditions; scale bars, 200 μm).

To further assess the function of CmHY5 in graft healing, we transiently silenced CmHY5 in melon scions using virus-induced gene silencing (TRV-CmHY5). RT-qPCR confirmed reduced CmHY5 transcript levels in silenced plants (Fig. 6a). Expression levels of CmWRKY23, CmWRKY69, and *CmGH9B3* were also reduced in TRV-CmHY5 scions compared to the control (Fig. 6b–d). Acid fuchsin staining showed delayed vascular connectivity in TRV-CmHY5 grafted seedlings (Fig. 6e). Histological sections revealed less callus formation and weaker vascular bundle development at the graft junction in silenced plants (Fig. 6f). These observations support a role for CmHY5 in promoting graft union healing, potentially through activation of CmWRKY23, CmWRKY69, and downstream *CmGH9B3* expression.

## Discussion

Efficient healing of the graft union is essential for the survival and vitality of grafted melon seedlings [1]. In this study, we characterized a light-regulated transcriptional module in which LED red light enhances graft union healing by activating CmHY5, which in turn promotes the expression of CmWRKY23, CmWRKY69, and downstream *CmGH9B3*. Our findings provide mechanistic insight into how environmental light cues are transduced into transcriptional regulation of cell wall remodeling genes that facilitate vascular reconnection.

Previous transcriptomic analyses suggested that *CmGH9B3*, encoding a β-1,4-glucanase, plays a role in the graft healing process of melon scions grafted onto squash rootstocks [21]. We confirmed this by showing that overexpression of *CmGH9B3* enhanced physical adhesion and vascular connectivity, while RNAi and TRV-mediated silencing delayed these processes. These results are consistent with studies in other species, such as tobacco and petunia, where GH9B3 homologs promote cell wall loosening and intercellular adhesion during graft formation [4, 6].

All organisms utilize transcriptional regulation as a fundamental process to modulate gene expression in accordance with internal and external stimuli [22–24]. However, only a limited number of WRKYs have been recognized as significant elements in the graft union healing process. One example is PlWRKY41a, which interacts directly with PlMYB43 to establish a protein complex. Consequently, this complex triggers the expression of *PlXTH4*, contributing to improved stem strength through the regulation of secondary cell wall thickness [25]. Research has identified that transcription factor WRKY53 of *Oryza sativa* negatively increased cell wall thickness, thus showing sensitivity to *Xanthomonas oryzae* pv. *oryzae* [26]. There are few reports on other WRKY transcription factors regulating plant secondary cell wall thickness and their relevance to graft union healing. We found that red light increased *CmGH9B3* transcript levels and improved graft healing phenotypes. The nuclear localization of *CmGH9B3*, along with its responsiveness to red light, prompted us to investigate upstream transcriptional regulators. Promoter analysis revealed multiple W-box motifs, and both CmWRKY23 and CmWRKY69 were identified as candidate transcription factors based on expression patterns and binding activity. Using Y1H, GUS, LUC, and EMSA assays, we demonstrated that CmWRKY23 and CmWRKY69 bind directly to the *CmGH9B3* promoter and activate its expression. Silencing of either transcription factor reduced *CmGH9B3* expression and delayed vascular reconnection, confirming their functional importance.

In addition to regulating *CmGH9B3*, CmWRKY23 and CmWRKY69 were found to interact physically, as shown by Y2H, LCI, and pull-down assays. Protein complex modeling using AlphaFold3 further identified key docking residues within the interaction interface. The findings indicate a potential functional transcriptional complex involving CmWRKY23 and CmWRKY69 for the co-regulation of *CmGH9B3* expression.

Furthermore, HY5 serves as a key regulator in orchestrating light signaling. Prior research has documented the binding of MdHY5 to the G-box motif within the MdMYBDL1 promoter, with the up-regulation of MdMYBDL1 resulting in heightened anthocyanin levels in healing apple tissues [27]. We also identified CmHY5 as an upstream light-responsive regulator of CmWRKY23 and CmWRKY69. CmHY5 transcript levels were elevated under red light, and its protein bound directly to the promoters of both WRKY genes. GUS, LUC, and EMSA assays confirmed that CmHY5 activated their transcription through G-box-like elements. Silencing of CmHY5 resulted in reduced expression of both WRKYs and *CmGH9B3*, and was associated with delayed graft healing. Together, these findings support a model in which red light induces CmHY5, which activates CmWRKY23 and CmWRKY69, leading to increased *CmGH9B3* expression and enhanced cell adhesion at the graft interface. Previous research confirmed that CmHY5 in the oriental melon scion can transport long distances to the squash rootstock, and induce the expression of CmoHY5. The interaction between CmHY5 and CmoHY5 positively regulated the expression of *CmoNRT2.1* to promote the nitrate uptake capacity of squash rootstock roots [28]. Under LED red light treatment conditions, the significantly up-regulated CmHY5 in melon scions is likely to be transported long distances to the rootstock to induce CmoHY5 expression. However, whether CmoHY5 directly or indirectly regulates *CmoGH9B3* expression to enhance the cell adhesion capacity of rootstock and promote graft union healing efficiency requires further experimental investigation.

Light quality is a crucial environmental signal that regulates diverse developmental processes in plants, including photomorphogenesis, hormone signaling, and stress responses [11, 14]. While previous work has linked light to hormone dynamics during grafting [8], our study is among the first to delineate a light-driven transcriptional pathway that directly regulates a cell wall-modifying enzyme involved in graft union healing. The integration of HY5 and WRKY signaling in this context expands the functional landscape of these well-characterized transcription factors and offers a framework for improving grafting efficiency through targeted light manipulation.

**Figure 7:**
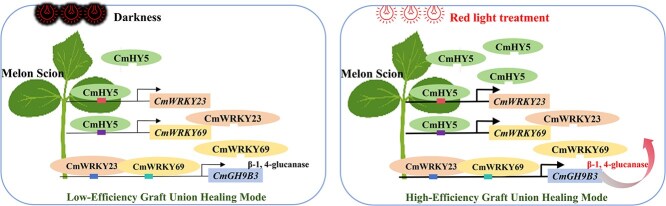
Proposed model of the red light-induced transcriptional module that promotes graft union healing in melon. LED red light activates the expression of CmHY5, which binds to and activates the promoters of CmWRKY23 and CmWRKY69. These WRKY transcription factors interact and activate the expression of *CmGH9B3*, a β-1,4-glucanase gene that enhances cell adhesion and vascular reconnection at the graft interface.

In conclusion, we propose a regulatory model in which LED red light enhances graft union healing by activating the CmHY5-CmWRKY23/69*-CmGH9B3* signaling cascade (Fig. 7). This model illustrates how environmental light cues can modulate transcriptional regulators that govern cell adhesion and vascular reconnection at the graft interface. Overall, our work provides a mechanistic understanding of light-mediated graft healing and offers practical strategies for improving grafting outcomes in cucurbits and potentially other horticultural crops.

## Materials and methods

### Plant materials and methods for grafting

Oriental melon (*C. melo* var. *makuwa* Makino, cv. ‘HuaBao’) and squash (*Cucurbita moschata*, cv. ‘ShengZhen No. 1’) were utilized as the scion and rootstock, respectively. Individual seeds were planted in cavity trays containing a substrate blend of peat, vermiculite, and perlite at a ratio of 2:1:1 (v/v/v). Grafting was conducted via the one-cotyledon technique once the first true leaf of the scion had completely developed [29]. The scion stem was cut diagonally at 45°C with a sharp blade, and part of the stem and roots were removed. One cotyledon of the rootstock was removed with a double-sided blade. Then, the two wounds were attached together and fixed with a grafting clamp. Grafted seedlings were kept in complete darkness for the first 2 DAG, followed by alternating black and white light conditions. Stem tissues were collected from scions at 2, 3, 4, 6, and 9 DAG from seedlings exhibiting uniform growth. Three biological replicates for each treatment were rapidly frozen in liquid nitrogen and preserved at −80°C. *Nicotiana benthamiana* plants were grown in pots containing standard potting medium in a controlled growth chamber maintained at 25°C with a 16-h light/8-h dark photoperiod.

### LED red light treatment

Red light treatment was applied starting at 2 DAG. Grafted seedlings were illuminated daily for 90 min (9:00–10:30 a.m.) using LED panels emitting at 660 nm (YPF = 79.1 μmol·m^−2^·s^−1^) under ambient temperature (23 ± 2°C). The control plants were subjected to dark conditions. Treatment duration was optimized based on *CmGH9B3* expression levels and visual assessment of scion wilting (Fig. S2). All other environmental conditions followed standard practices for grafted seedling cultivation.

### Cell wall thickness analysis

Samples were fixed in 4% paraformaldehyde to maintain quality, trimmed, dehydrated, embedded, sectioned, stained, sealed, and examined under a microscope following our institutional pathology laboratory's standard operating procedures (SOPs). Cell wall thickness was measured at five locations in each image using Media Cybernetics' Image-Pro Plus 6.0 software, with the average value calculated in millimeters.

### Calculation of physical adhesion and vascular connectivity

At 2 DAG, the grafting clips were uniformly removed, and the number of dropped and adhered scions among 100 grafted seedlings was tallied individually. The physical adherence rate was determined by dividing the number of adhered scions by 100. Subsequently, at 6 DAG, the xylem connections of scions in 50 grafted seedlings were visualized using acid fuchsin. Stem segments (1 cm) above and below the graft union were sectioned, and the rootstock stems were vertically immersed in a 1% acid fuchsin solution for 1 h. Cross-sections of 2.5-mm segments above the graft union were then examined for dye uptake. The uptake of acid fuchsin was visualized and documented using a confocal laser scanning microscope. Furthermore, the conduit connectivity rate was calculated by dividing the number of scions absorbing fuchsin by 50. Each experimental treatment was replicated three times.

### Histological section observation

For the purpose of anatomical examination of the graft interface, stem segments spanning the graft junction, measuring 0.5–1.0 cm, were preserved in FAA solution (70% ethanol/glacial acetic acid/formaldehyde = 90:5:5) [30]. Following vacuum infiltration, the samples were stored at 4°C and subjected to dehydration through a series of graded ethanol solutions. For dehydration, plant tissue was immersed in 30%, 50%, 75%, 80%, 90%, and 100% gradient ethanol. Leica paraffin wax was used for embedding, a German Leica slicer was used for cross-cutting tissue, a water bath was used to spread the film, and a dryer was used to dry the adhesive film. The slices were successively dewaxed and rehydrated in different proportions of ethanol and xylene solution. Moreover, the slices were sealed by staining them with Senna Red Solid Green (or Toluidine Blue) and then stained. The connection of the grafted joint was observed and imaged by a fluorescence microscope.

### Extraction of RNA, synthesis of cDNA, and analysis of gene expression

Melon stem tissue was subjected to liquid nitrogen grinding for total RNA extraction, followed by purification using the Ultrapure RNA Kit (CWBIO, China) as per the manufacturer’s guidelines. RNA quality and integrity were assessed by electrophoresis on a 1.0% agarose gel, and purity was determined by measuring absorbance at 260 nm. Utilizing the PrimeScript™ RT Master Mix (Takara, Dalian, China), first-strand cDNA was synthesized from 1000 ng of total RNA and subsequently used as the template for RT-qPCR analysis. RT-qPCR was performed with SuperReal PreMix Plus (SYBR Green) (Takara, Dalian, China) on an ABI PRISM 7500 real-time PCR system (Applied Biosystems, Thermo Fisher Scientific, USA) according to the manufacturer’s protocol. Design of gene-specific primers was accomplished through Primer-BLAST, and their sequences are detailed in Table S1. Quantification of gene expression levels was carried out utilizing the 2^−ΔΔCt^ method, with melon 18S rRNA as the internal control. All experimental procedures were replicated three times biologically and three times technically.

### Subcellular localization

The complete coding sequence of *CmGH9B3*, devoid of a stop codon, was integrated into the pCAMBIA1300 vector to produce the hybrid expression vector 35S::*CmGH9B3*::GFP. The construct was subsequently inserted into *Agrobacterium tumefaciens* strain GV3101 and transferred into *N. benthamiana* leaves through infiltration. Subcellular localization was evaluated using a fluorescence confocal microscope (TCS SP8; Leica, Wetzlar, Germany) [31]. Detection of green fluorescence occurred at an excitation wavelength of 488 nm and emission range of 520–540 nm, while red fluorescence was noted at an excitation wavelength of 561 nm and emission range of 610–630 nm. All fluorescence imaging experiments were carried out using no fewer than three biological replicates. The primers employed for construct amplification are listed in Table S2.

### Virus-induced gene silencing assay

PCR amplified the *CmGH9B3*, CmWRKY23, CmWRKY69, and CmHY5 gene, cloned it into the TRV2 vector, and constructed the TRV2-*CmGH9B3*, TRV2-CmWRKY23, TRV2-CmWRKY69, and TRV2-CmHY5 silencing vector (Table S2) [32]. These plasmids were subsequently transformed into the Agrobacterium tumefaciens GV3101 strain. The pTRV1 was mixed 1:1 with TRV2-*CmGH9B3*, TRV2-CmWRKY23, TRV2-CmWRKY69, and TRV2-CmHY5, respectively, and similarly, pTRV1 was mixed 1:1 with pTRV2 as a control and injected into the abaxial surface of melon cotyledons. When the oriental melon scion seedlings’ cotyledons had fully opened, the infective liquid was injected into the oriental melon scion cotyledon with the syringe to obtain TRV2-*CmGH9B3*, TRV2-CmWRKY23, TRV2-CmWRKY69, TRV2-CmHY5, and control melon strains. Following infiltration, the plants were placed in a growth chamber under dark conditions for 18 h, then subjected to regular light/dark cycles (26°C light period, 18°C dark period). Grafting was conducted upon full expansion of one true leaf, with samples collected at 2 to 9 DAG. The primers utilized for construct amplification can be found in Table S2.

### *Agrobacterium tumefaciens* experiment

The full coding sequence of *CmGH9B3* lacking a stop codon was inserted into the pCAMBIA1300 vector to create the chimeric expression vector 35S::*CmGH9B3*::GFP. Subsequently, the constructed vector with 35S::GFP empty was transformed into K599 *Agrobacterium* competent cells. The bacteria were shaken to obtain 1300-*CmGH9B3* and 1300 empty vector infiltration solution. When the oriental melon scion seedlings cotyledons had fully opened, melon stems were cut diagonally at 45° angle and placed in 1 ml of infestation solution for 30 min, then placed in new cavity trays to grow individually (peat/vermiculite: perlite = 2:1:1) and injected with 1 ml of infection solution around them. After infestation, they were incubated in an artificial light incubator with 72 h of darkness followed by regular day–night alternation at 26°C for light and 18°C for dark and a humidity of 85%. After 2 weeks, the roots were tested for green fluorescence using an excitation wavelength of 488 nm for GFP, resulting in *CmGH9B3*-OE lines and WT melon lines. Melon seedlings with green fluorescence signal were grafted, and samples were taken from 0 to 9 DAG after grafting. The primers utilized for construct amplification can be found in Table S2.

### Stable transformation of oriental melon

The *CmGH9B3*-RNAi construct, targeting *CmGH9B3*, was created by inserting a *CmGH9B3* gene fragment into the pKANNIBAL vector. This plasmid was then transferred into *A. tumefaciens* for the genetic modification of oriental melon using cotyledon explants from the T0948 inbred line. Transgenic experiments utilized T1 generation seeds, with kanamycin-resistant plants being selected for subsequent RT-qPCR analysis to evaluate gene silencing efficacy [29]. The media composition used in the transformation and selection processes is detailed in Tables S3–S5.

### Y1H assay

The pAbAi vector was utilized to clone the promoters of *CmGH9B3*, CmWRKY23, and CmWRKY69, resulting in the creation of *CmGH9B3*pro-pAbAi, *CmWRKY23*pro-pAbAi, and *CmWRKY69*pro-pAbAi constructs. Following this, the pGADT7 vector was employed to ligate the full-length sequences of CmWRKY23, CmWRKY69, and CmHY5, leading to the formation of CmWRKY23-pGADT7, CmWRKY69-pGADT7, and CmHY5-pGADT7 constructs. The Y1H assay was conducted by co-transforming different combinations of bait (pAbAi) and prey (pGADT7) vectors into yeast cells. The interactions were assessed on selection media containing Aureobasidin A (AbA) at 50, 75, 100 and 150 ng/ml (Warbio, China) [33]. The primer sequences utilized in the Y1H assay can be found in Table S2.

### GUS and luciferase reporter assays

Promoter regions from *CmGH9B3*, CmWRKY23, and CmWRKY69 were cloned into either pBI101 or pGreenII 0800-LUC vectors, while the CDS sequences of CmWRKY23, CmWRKY69, and CmHY5 were inserted into pRI101 or pCAMBIA1300. These constructs were then co-infiltrated into *N. benthamiana* leaves for GUS histochemical staining or luciferase imaging using a Berthold LB985 system. Relative expression levels were assessed by quantifying GUS enzymatic activity or LUC/REN luminescence ratios.

### Electrophoretic mobility shift assay

The coding sequences (CDS) of CmWRKY23, CmWRKY69, and CmHY5 were cloned and integrated into the pD2P-1.06eTM vector, where they were fused with a His-tag and GST-tag. These constructs were utilized for protein induction following the protocol provided by the BiYunTian kit. The probes were custom-synthesized by Sebastian Biological Company in Beijing, China. Biotin-labeled double-stranded DNA probes for EMSA were prepared by annealing complementary oligonucleotides. The oligonucleotides were denatured at 95°C for 5 min, annealed at 72°C for 20 min, and then cooled to room temperature. The biotin-labeled probes targeting the promoters of *CmGH9B3*, CmWRKY23, and CmWRKY69, as well as the coding sequences (CDS) of CmWRKY23, CmWRKY69, and CmHY5, were detailed in Table S2. EMSA was conducted using the LightShiftTM Chemiluminescent EMSA Kit (Cat. No. 20148, Thermo Scientific) following the manufacturer's protocol [34].

### Y2H assay

The CDSs of CmWRKY23 and CmWRKY69 were cloned into pGADT7 and pGBKT7 vectors separately. These constructs, along with empty vectors, were co-transformed into the Y2HGold yeast strain using the Y1H assay protocol. Following a 2- to 3-day incubation at 30°C, yeast colonies were then plated on DDO and QDO media supplemented with X-α-Gal and AbA (300 ng/ml) to evaluate protein–protein interactions.

### Luciferase complementary imaging assay

Gene-specific fragments were amplified by PCR and fused into firefly luciferase (LUC) complementation vectors. The resulting constructs (nLUC-CmWRKY69 and cLUC-CmWRKY23) were used for split-LUC assays. *Agrobacterium* strains harboring the constructs were simultaneously infiltrated into *N. benthamiana* leaves, positioning the interaction pair and control constructs on opposing sides of the main vein [35]. After incubation in darkness for 24 h followed by 48 h under low-light conditions, fluorescence signals were detected using a live imaging system (LB985, Berthold, Germany).

### Pull-down assay

CmWRKY69-GST and GST protein supernatants were initially introduced into the chromatography column to bind with ProteinIso^®^ GST Resin media following the GST protein purification protocol. The GST protein column served as the negative control. Subsequently, purified CmWRKY23-HIS protein was added to the aforementioned chromatography columns containing CmWRKY69-GST and GST, and then incubated at 4°C with 100 rpm for 1 h [36]. The chromatography column was washed 5–10 times with 1× PBS, and the proteins were finally eluted with GST elution buffer. The above eluted protein samples were subjected to WB detection using an anti-HIS antibody to determine whether or not there were interactions between the proteins.

### Protein–protein interaction modeling by AlphaFold3

We used AF3 for protein complex prediction, where AlphaFold is a deep learning system developed by DeepMind to predict the three-dimensional structure of proteins accurately [37]. Protein complex prediction was performed by inputting amino acid sequences, and after obtaining the result file, the optimal conformation cif0 file was used for binding site mapping, and the pymol software was used as the mapping tool.

### Statistical analysis

Statistical analyses were conducted using SPSS software (version 26.0), presenting data as means ± standard deviation (SD) derived from a minimum of three independent biological replicates. Distinctions among treatments were assessed through one-way analysis of variance (ANOVA), with significance set at *P* < 0.05 unless otherwise stated.

## Supplementary Material

Web_Material_uhaf251

## Data Availability

The data that support the findings of this study are available in the manuscript and Supporting Information of this article. The authors confirm that all experimental data are available and can be accessed through the main text and/or the supplemental data. The accession codes are listed as follows: *CmGH9B3* (MELO3C022160), CmWRKY23 (MELO3C015910), CmWRKY69 (MELO3C018717), and CmHY5 (MELO3C011250).
