# Supplementary material for: CmHY5-CmWRKY23/69-CmGH9B3 module mediates red light promoted graft union healing of melon grafted onto squash
Source: Hortic Res. 2025 Sep 17;13(1):uhaf251. doi: 10.1093/hr/uhaf251 (PMC12858254; doi:10.1093/hr/uhaf251)
Supplement: Web_Material_uhaf251 [file web_material_uhaf251.zip › Figure 7.pdf]

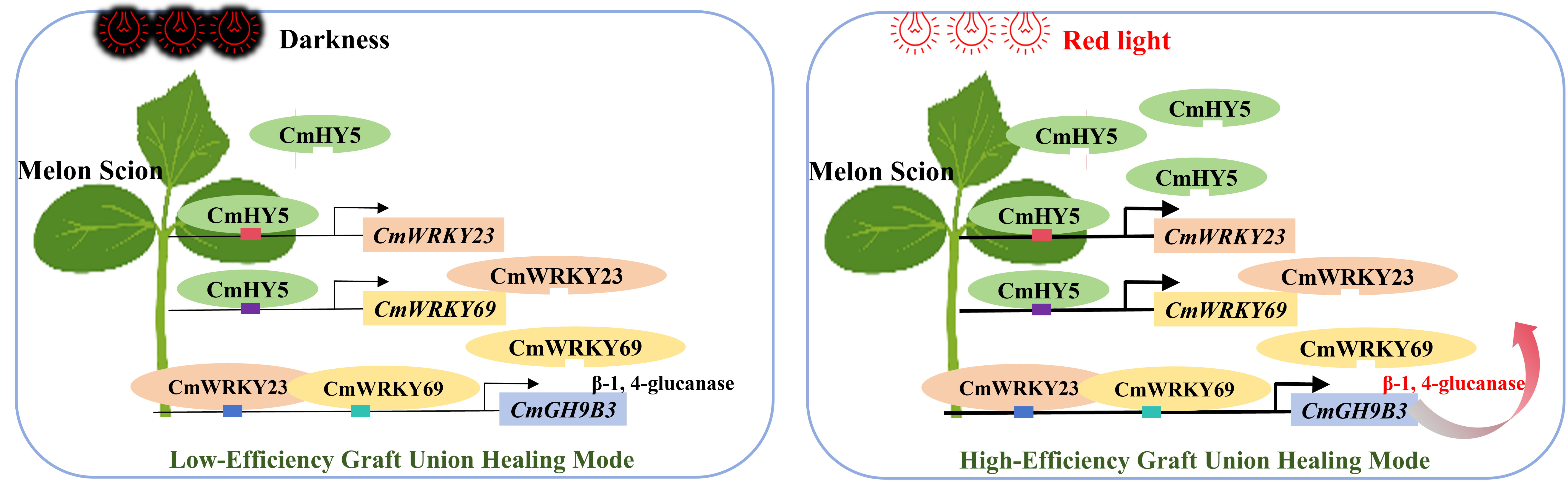

Darkness
Melon Scion
CmHY5
CmHY5
CmWRKY23
CmHY5
CmWRKY23
CmWRKY69
CmWRKY69
CmWRKY23
CmWRKY69
β-1, 4-glucanase
CmGH9B3
Low-Efficiency Graft Union Healing Mode
Red light
Melon Scion
CmHY5
CmHY5
CmHY5
CmHY5
CmWRKY23
CmHY5
CmWRKY23
CmWRKY69
CmWRKY69
CmWRKY23
CmWRKY69
β-1, 4-glucanase
CmGH9B3
High-Efficiency Graft Union Healing Mode
