# Supplementary figures and images for: CmHY5-CmWRKY23/69-CmGH9B3 module mediates red light promoted graft union healing of melon grafted onto squash
Source: Hortic Res. 2025 Sep 17;13(1):uhaf251. doi: 10.1093/hr/uhaf251 (PMC12858254; doi:10.1093/hr/uhaf251)

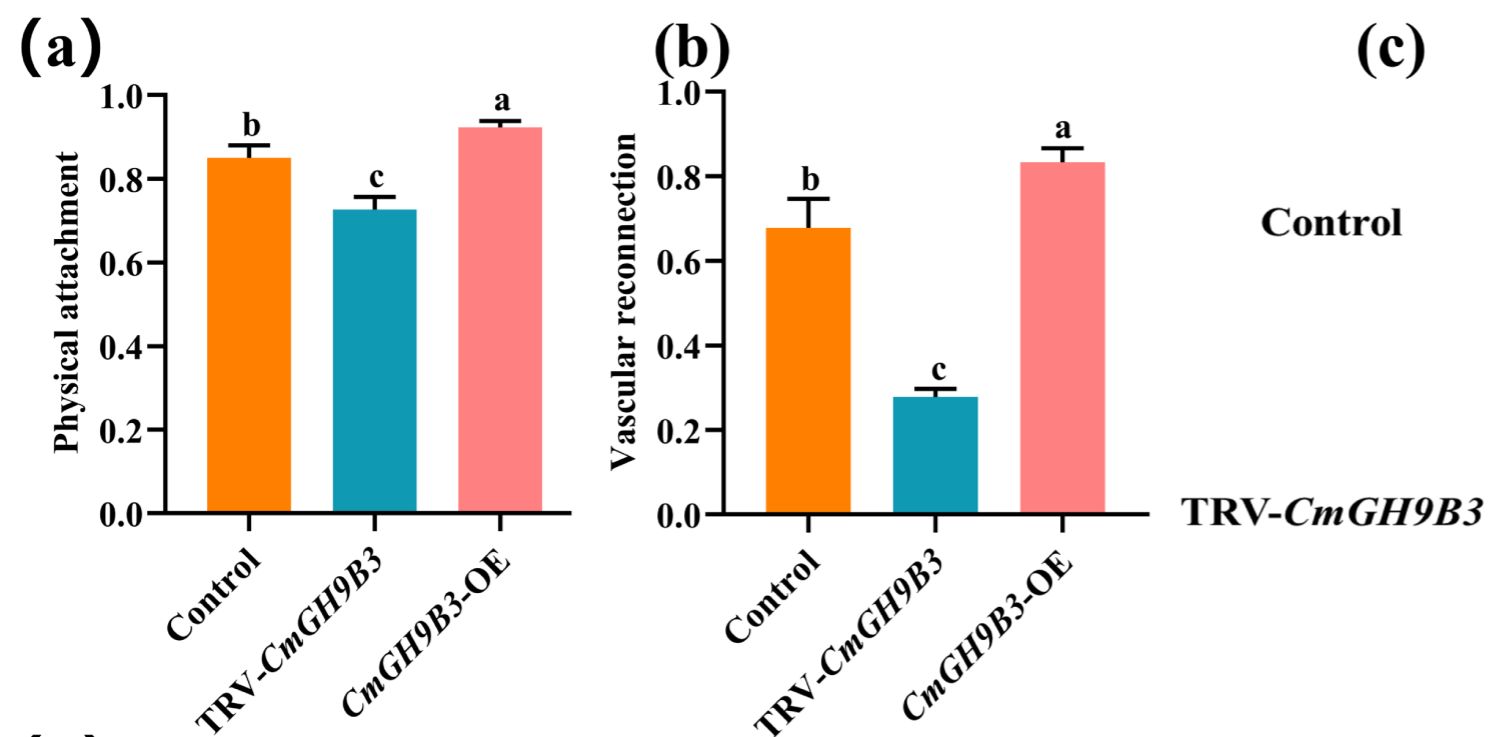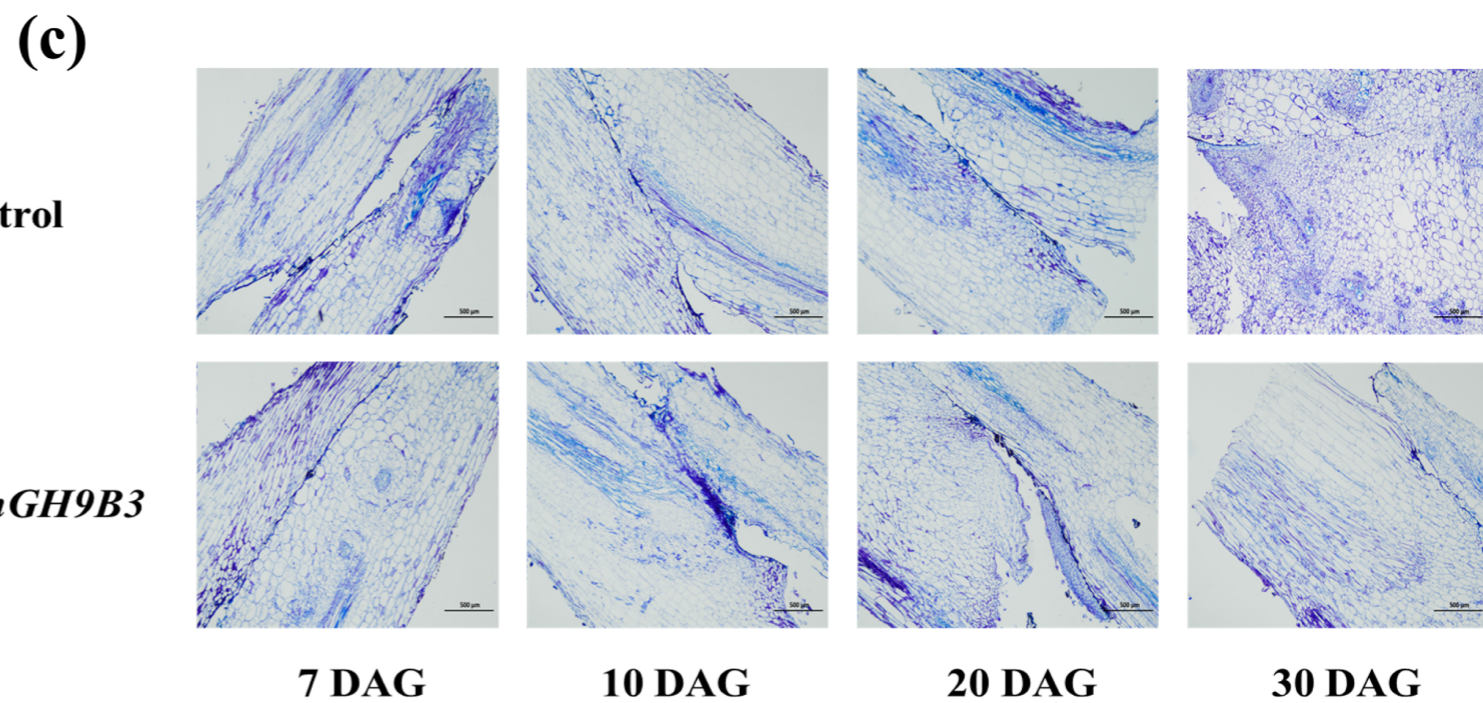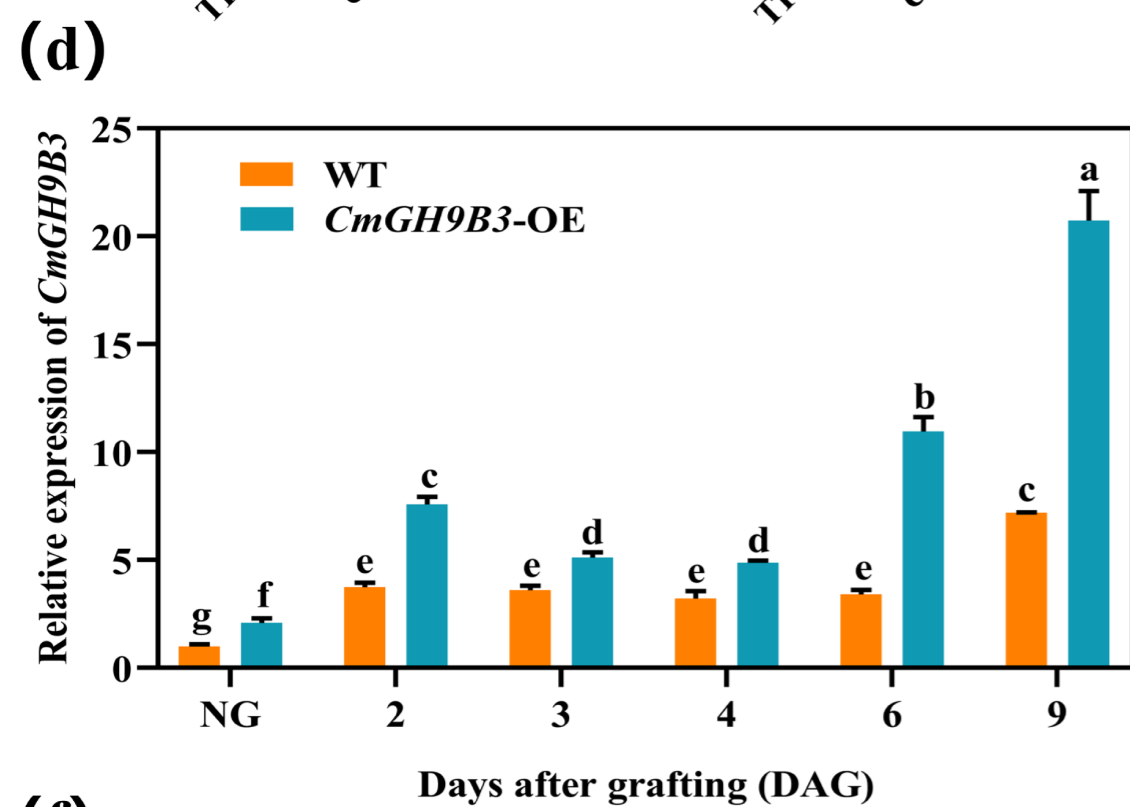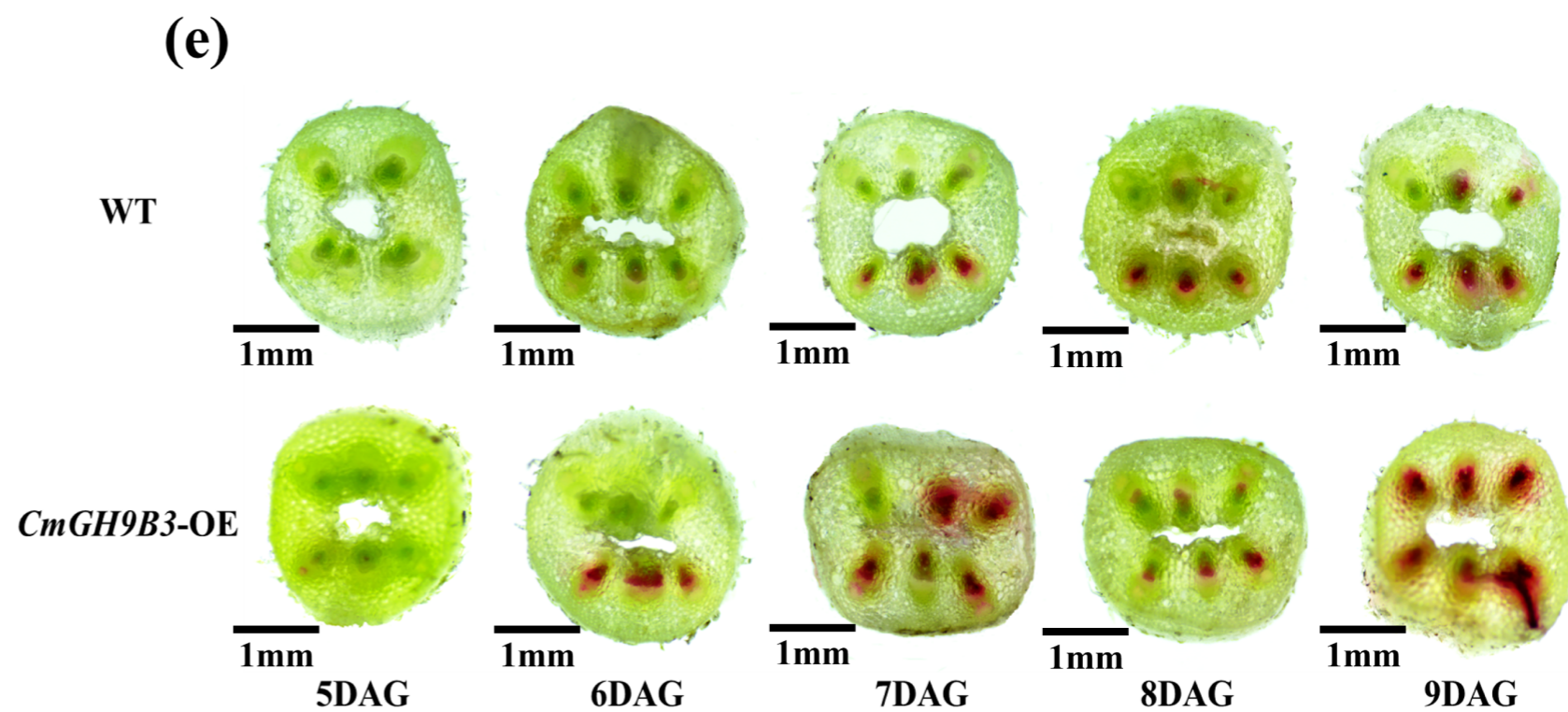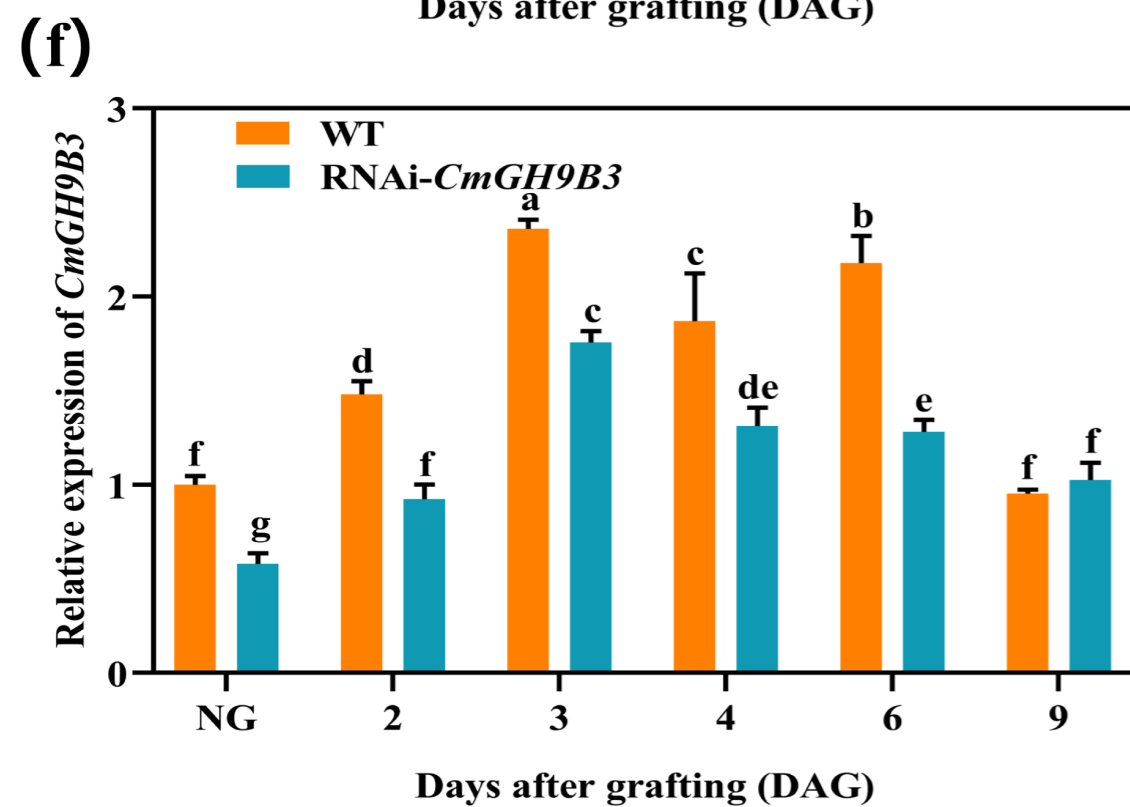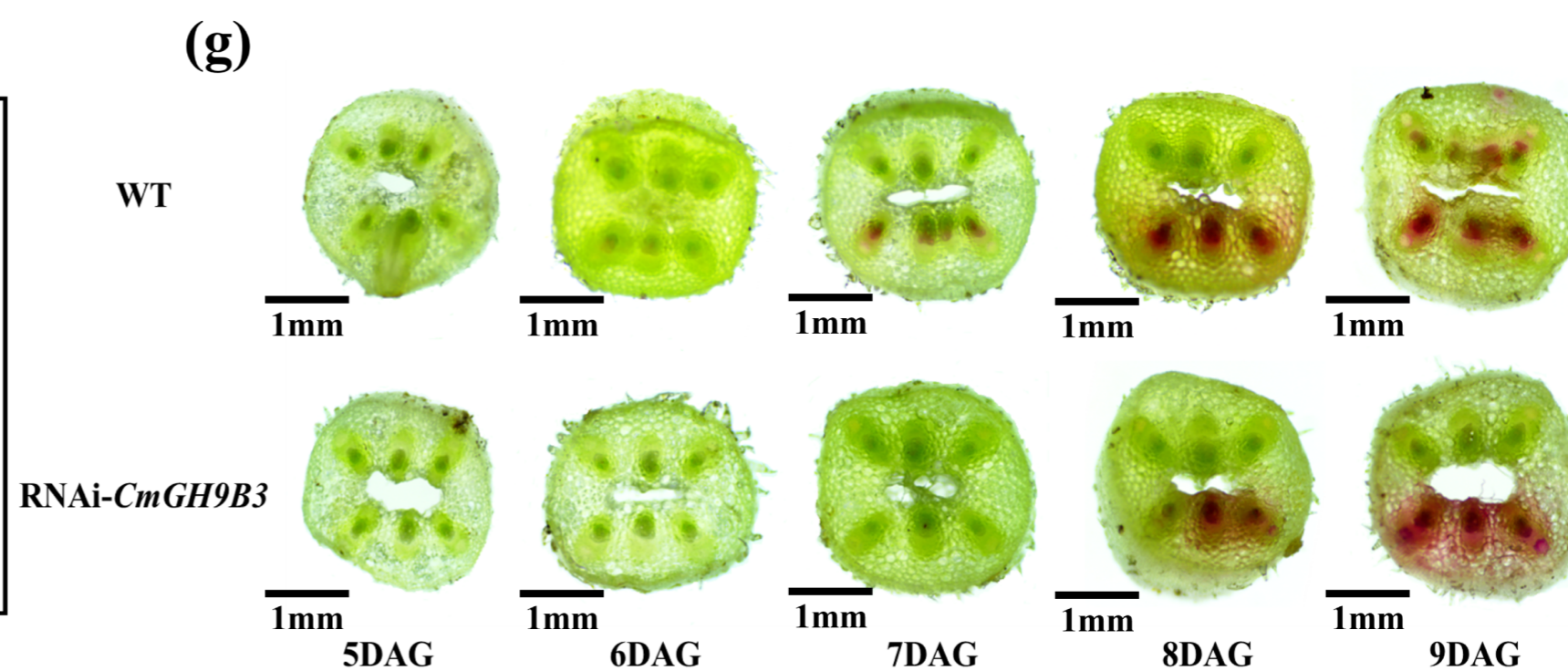

Supplement: Web_Material_uhaf251 [file web_material_uhaf251.zip › Figure 1.pdf]

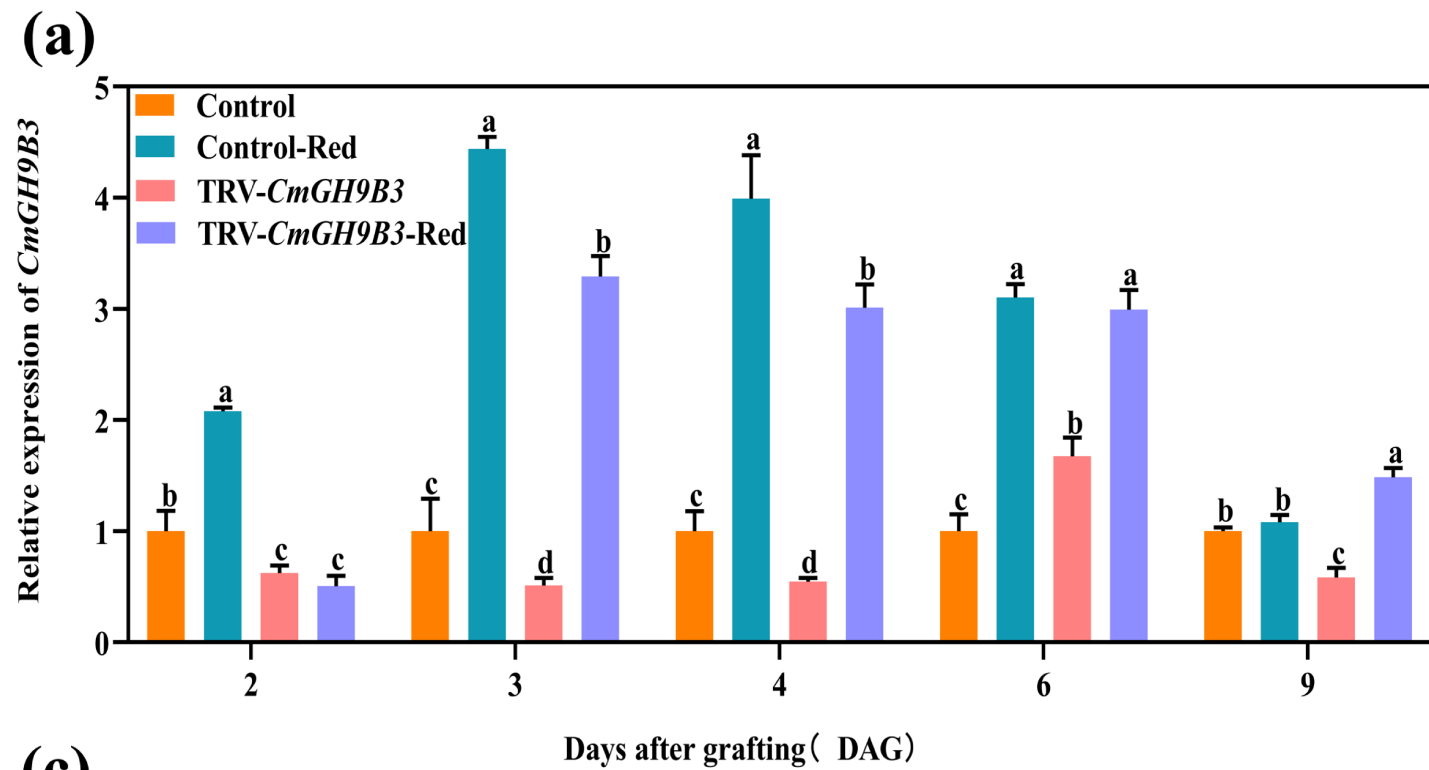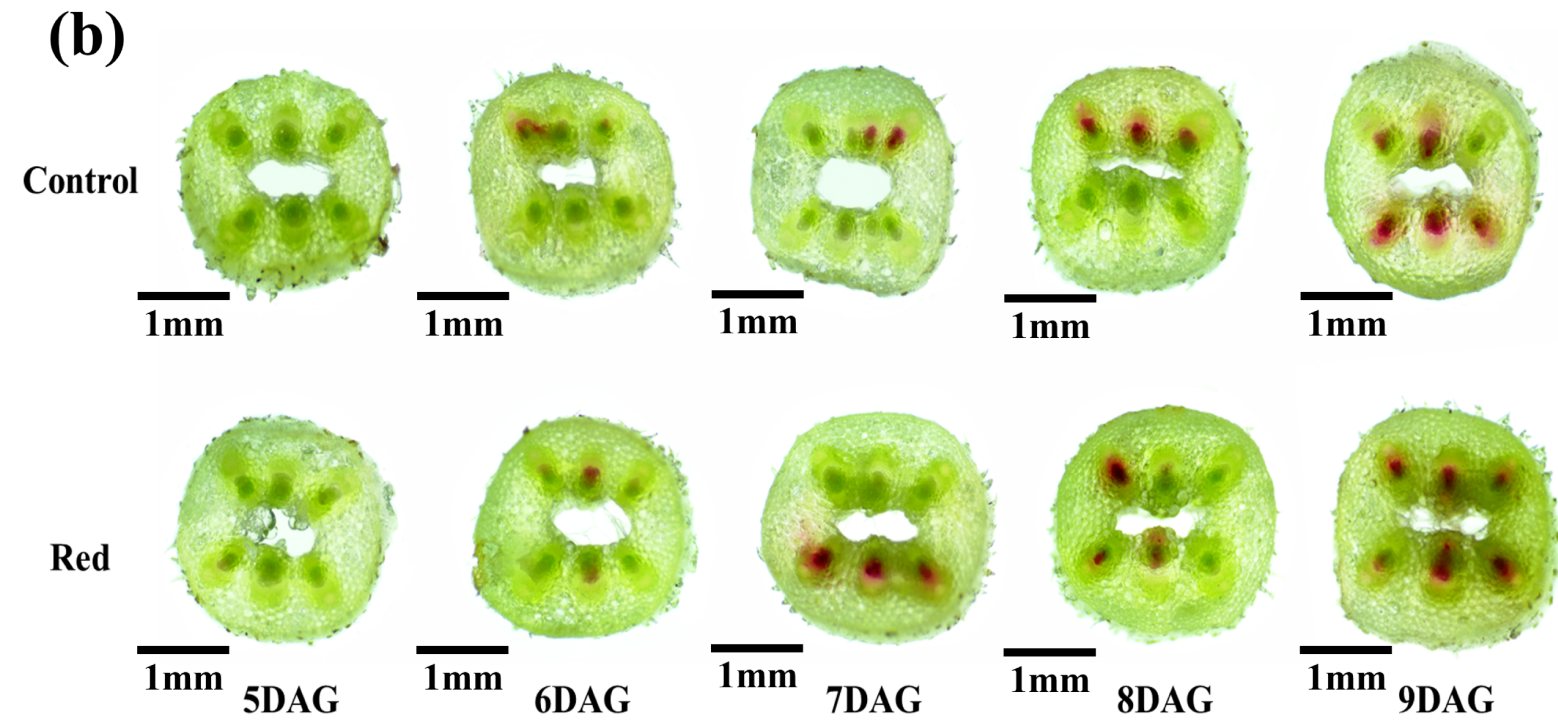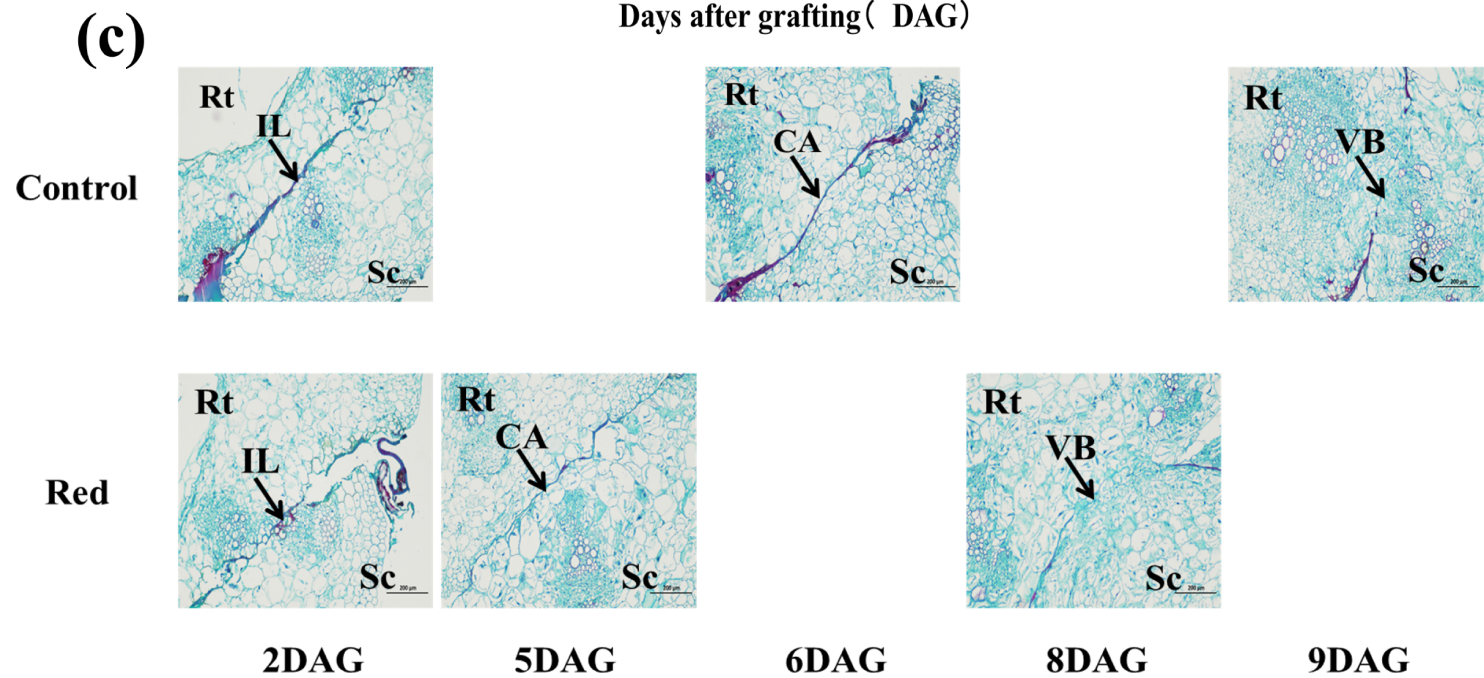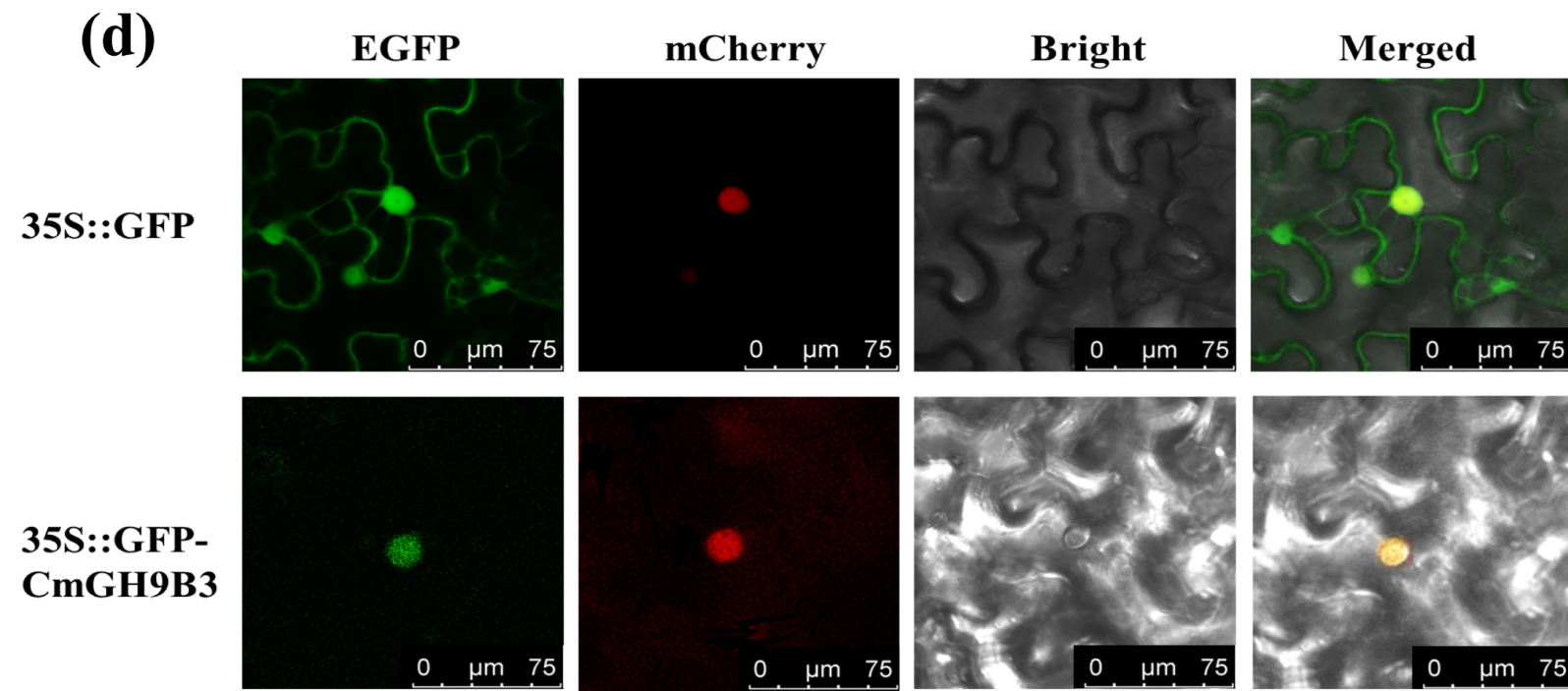

Supplement: Web_Material_uhaf251 [file web_material_uhaf251.zip › Figure 2.pdf]

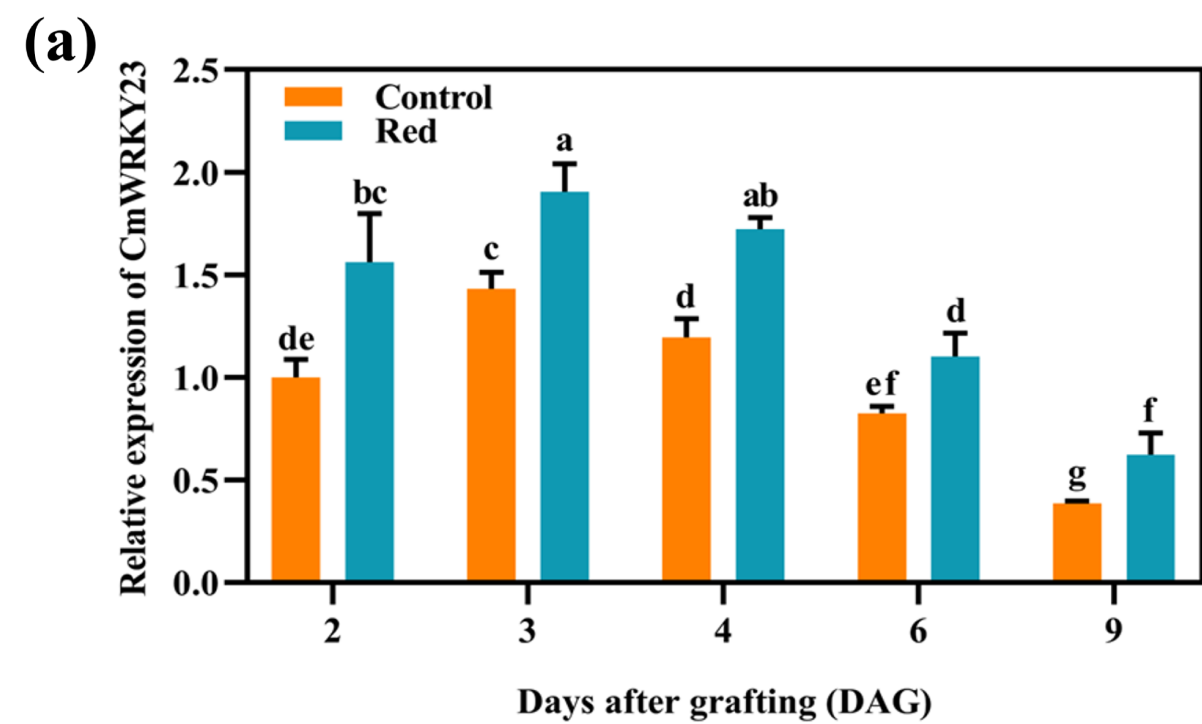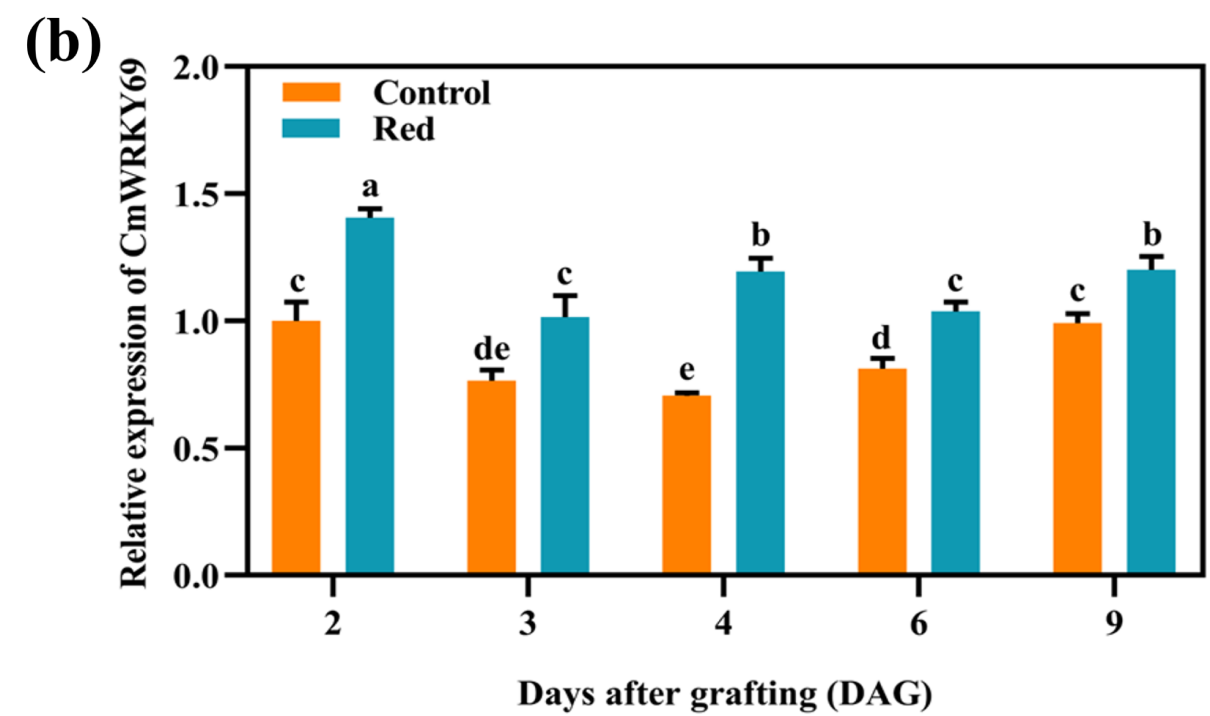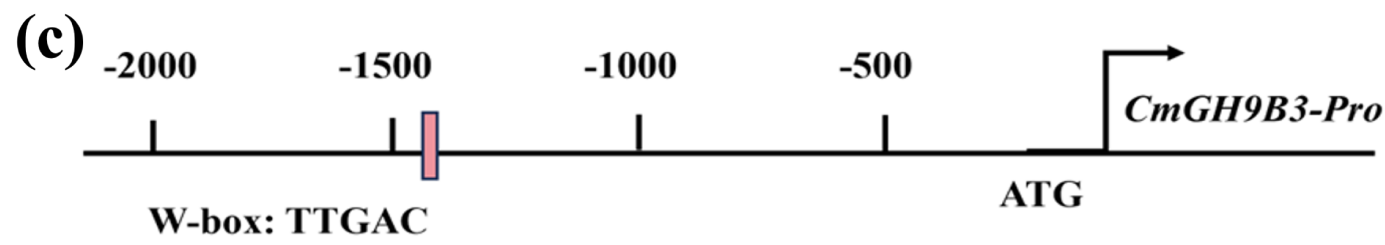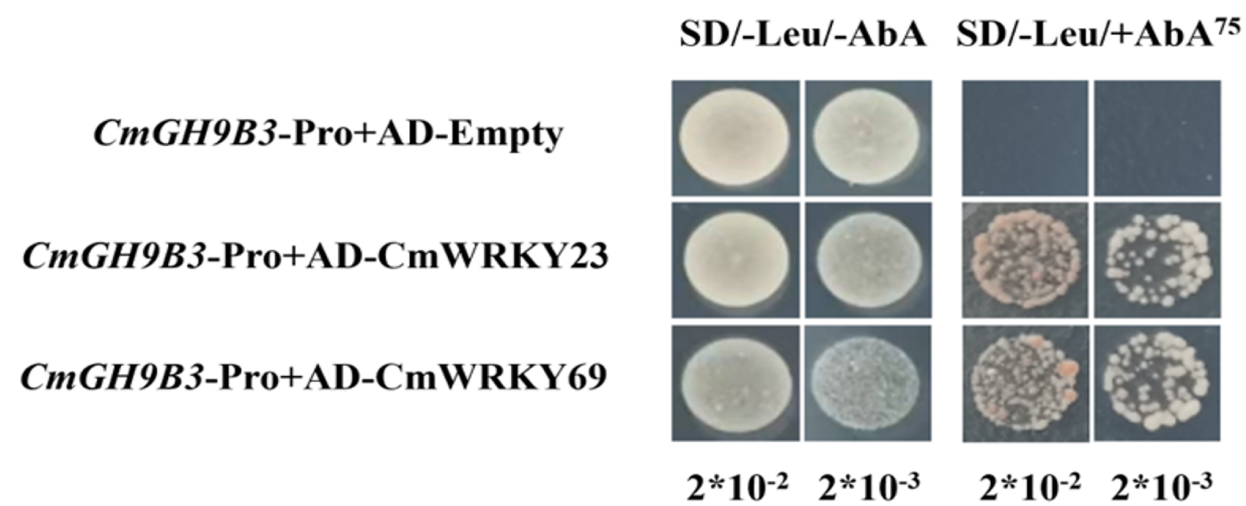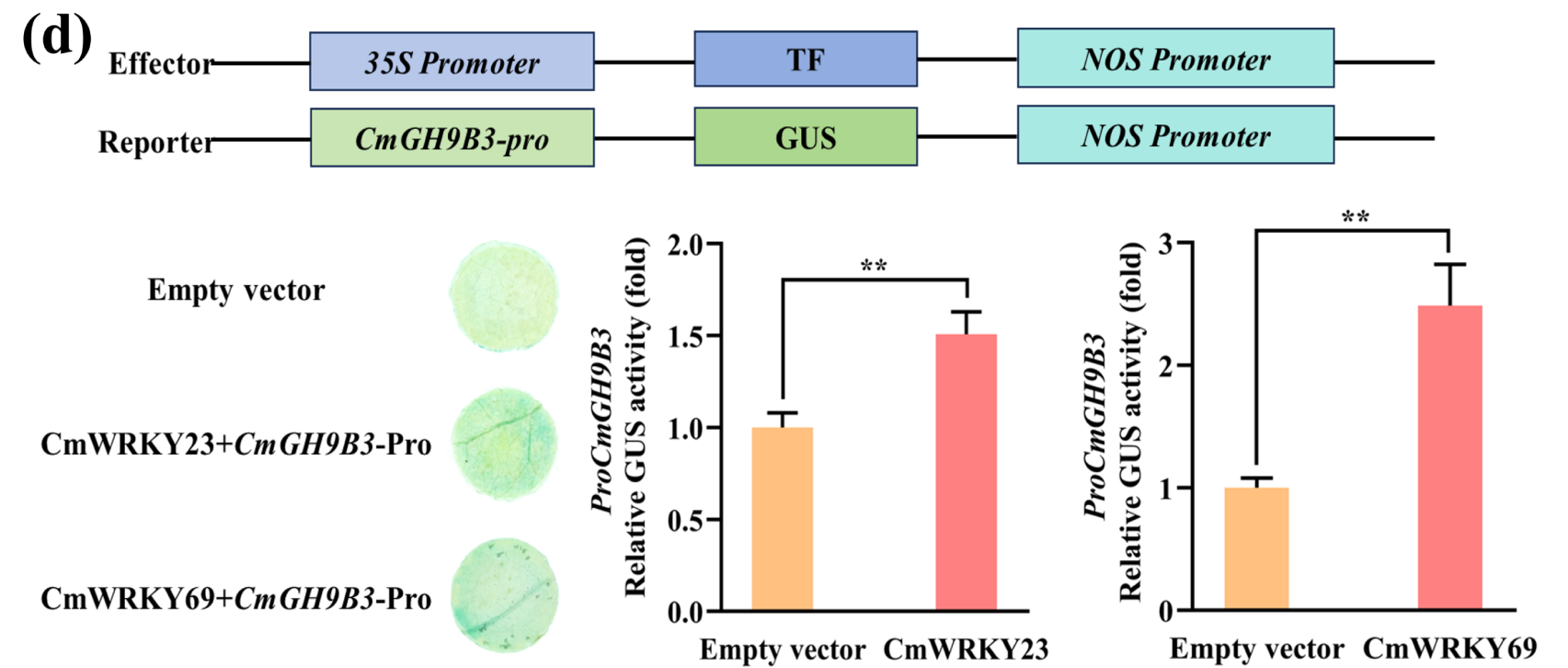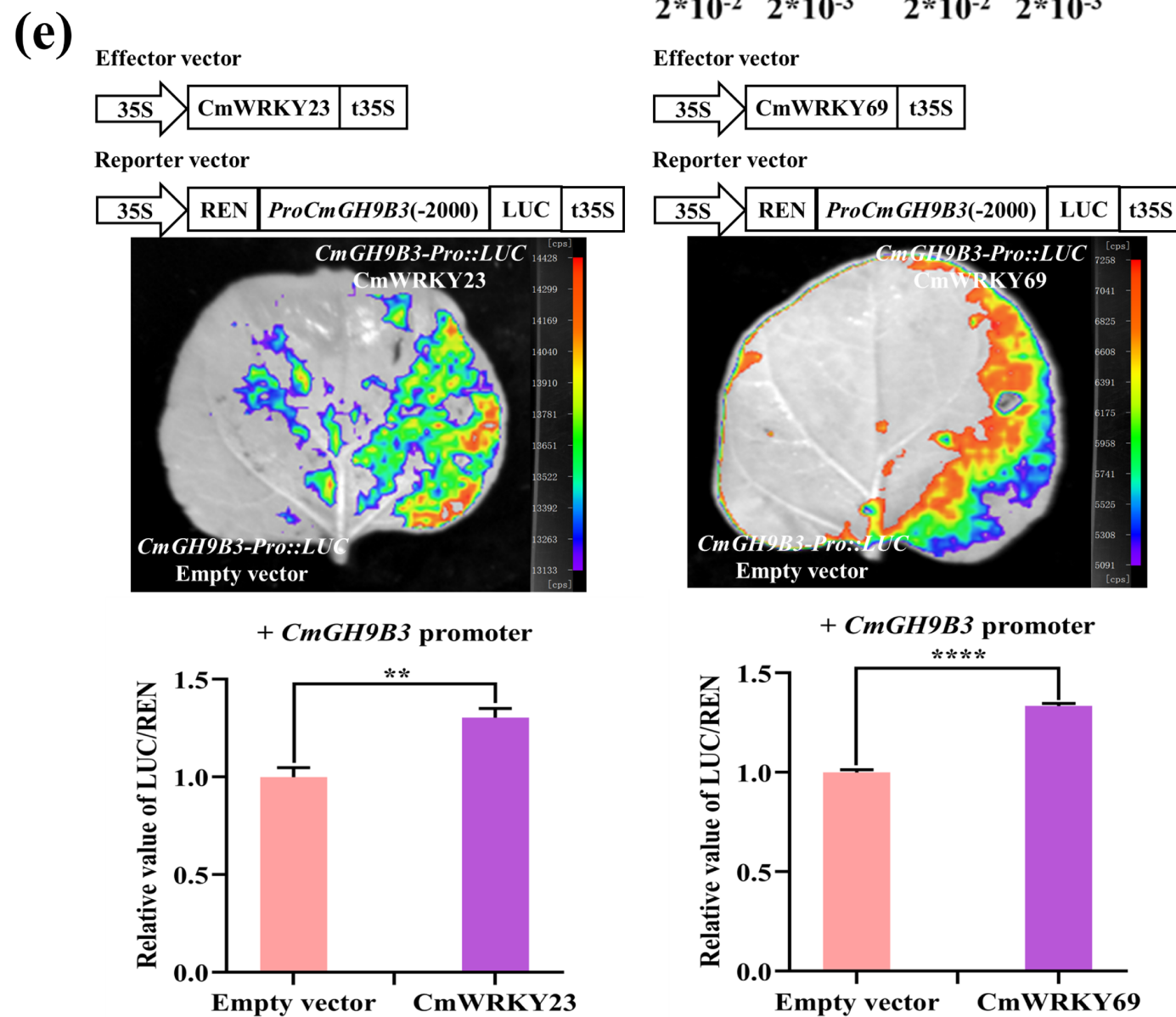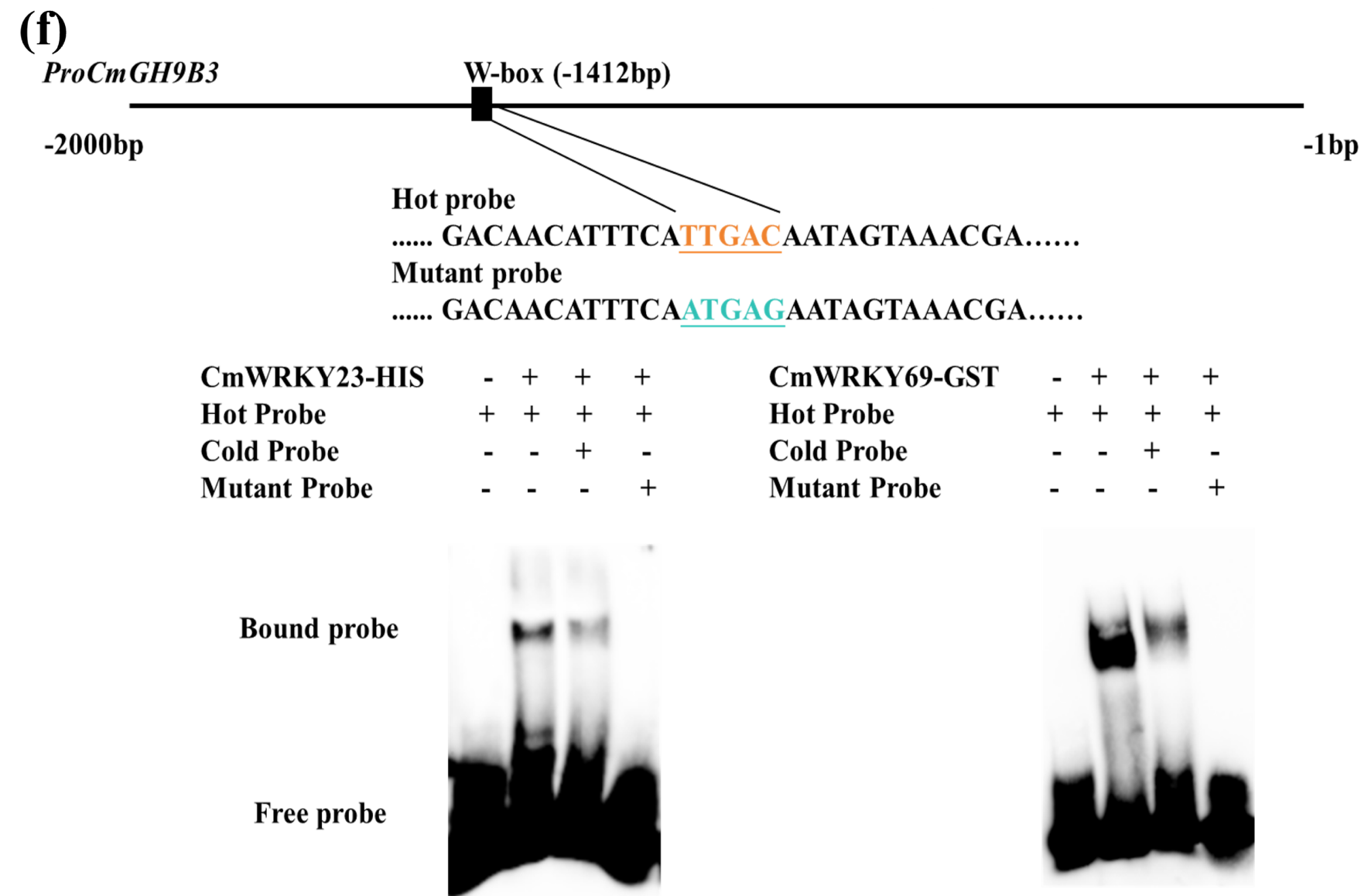

Supplement: Web_Material_uhaf251 [file web_material_uhaf251.zip › Figure 3.pdf]

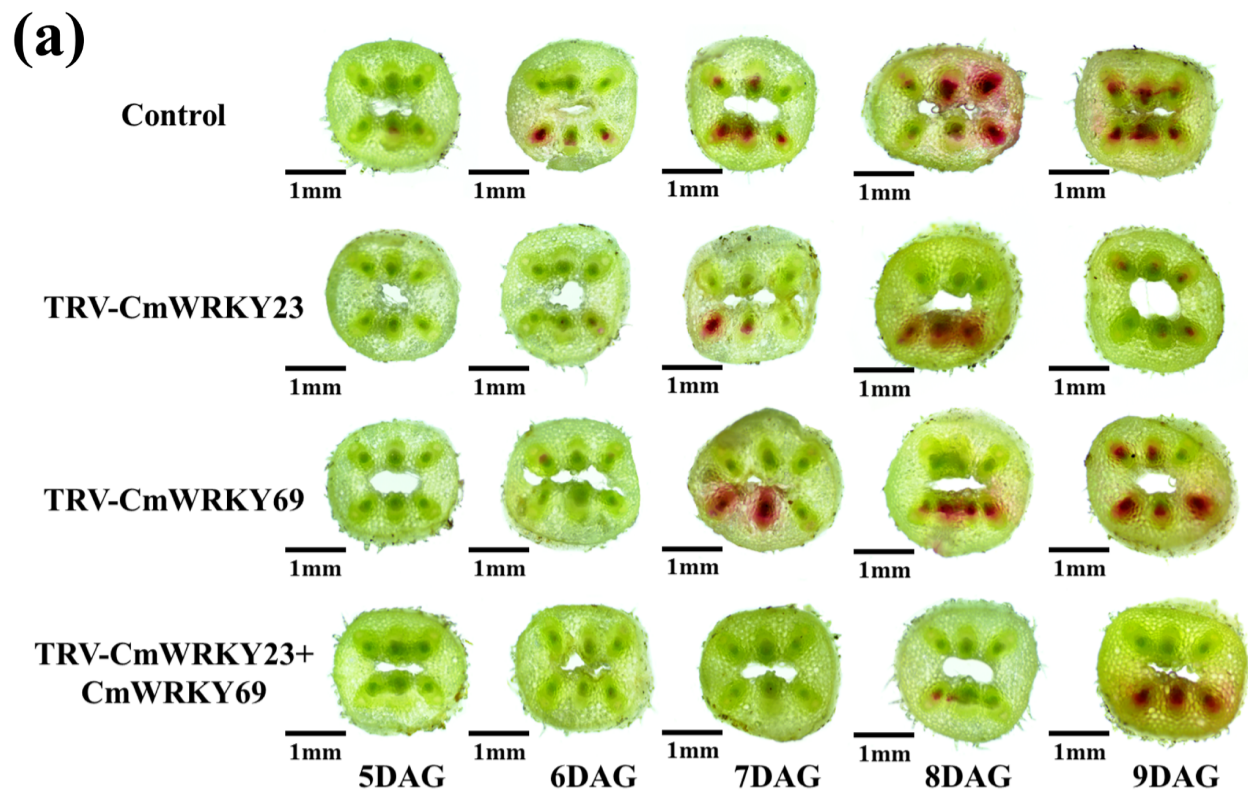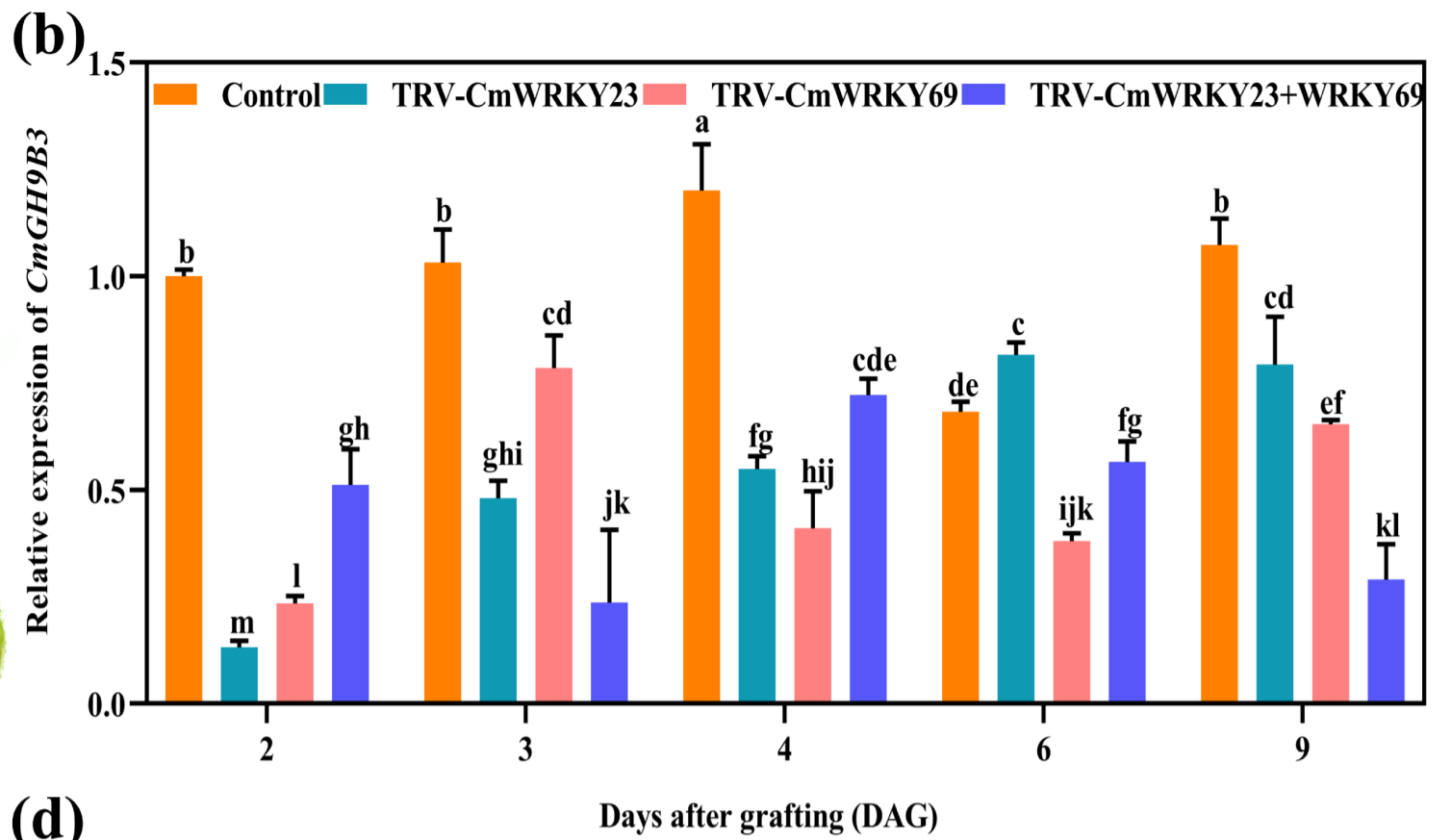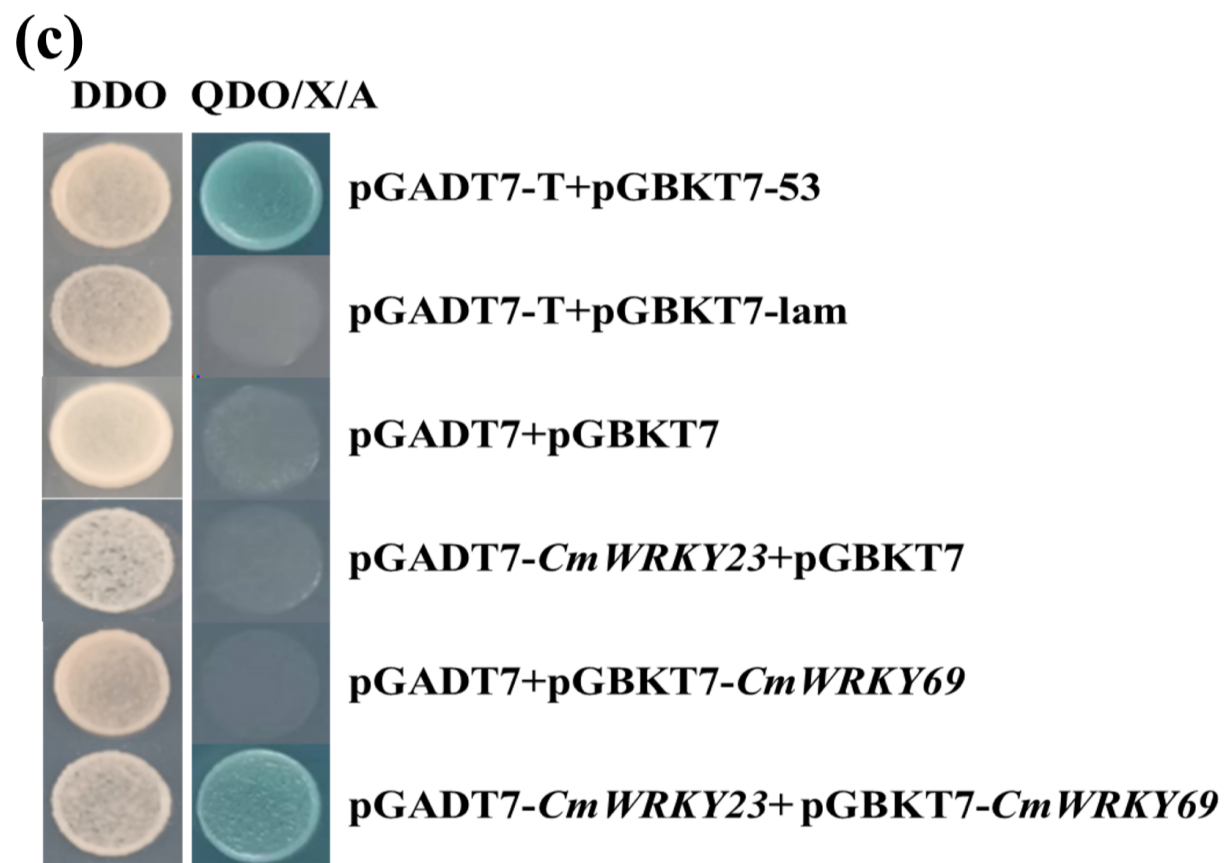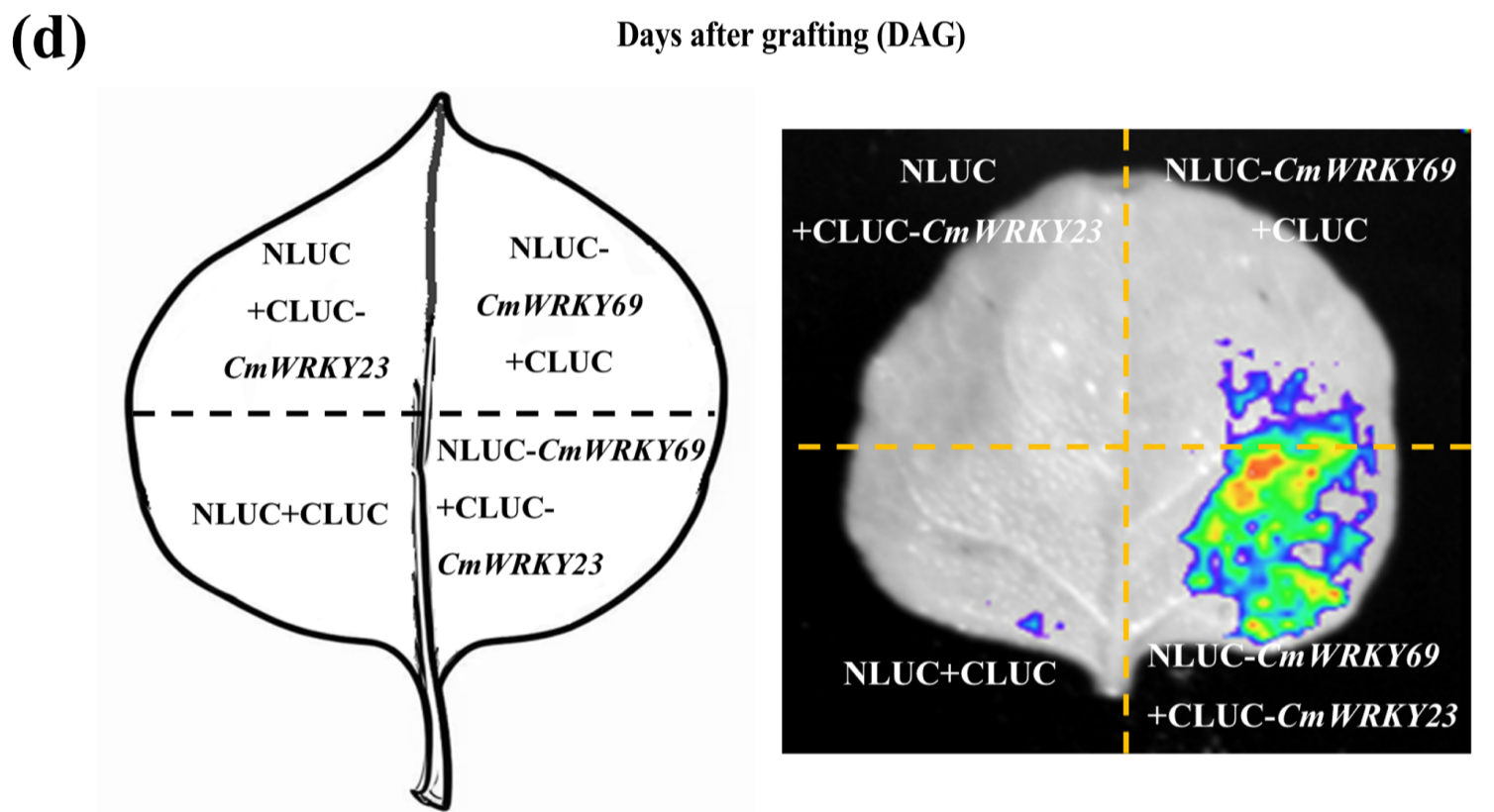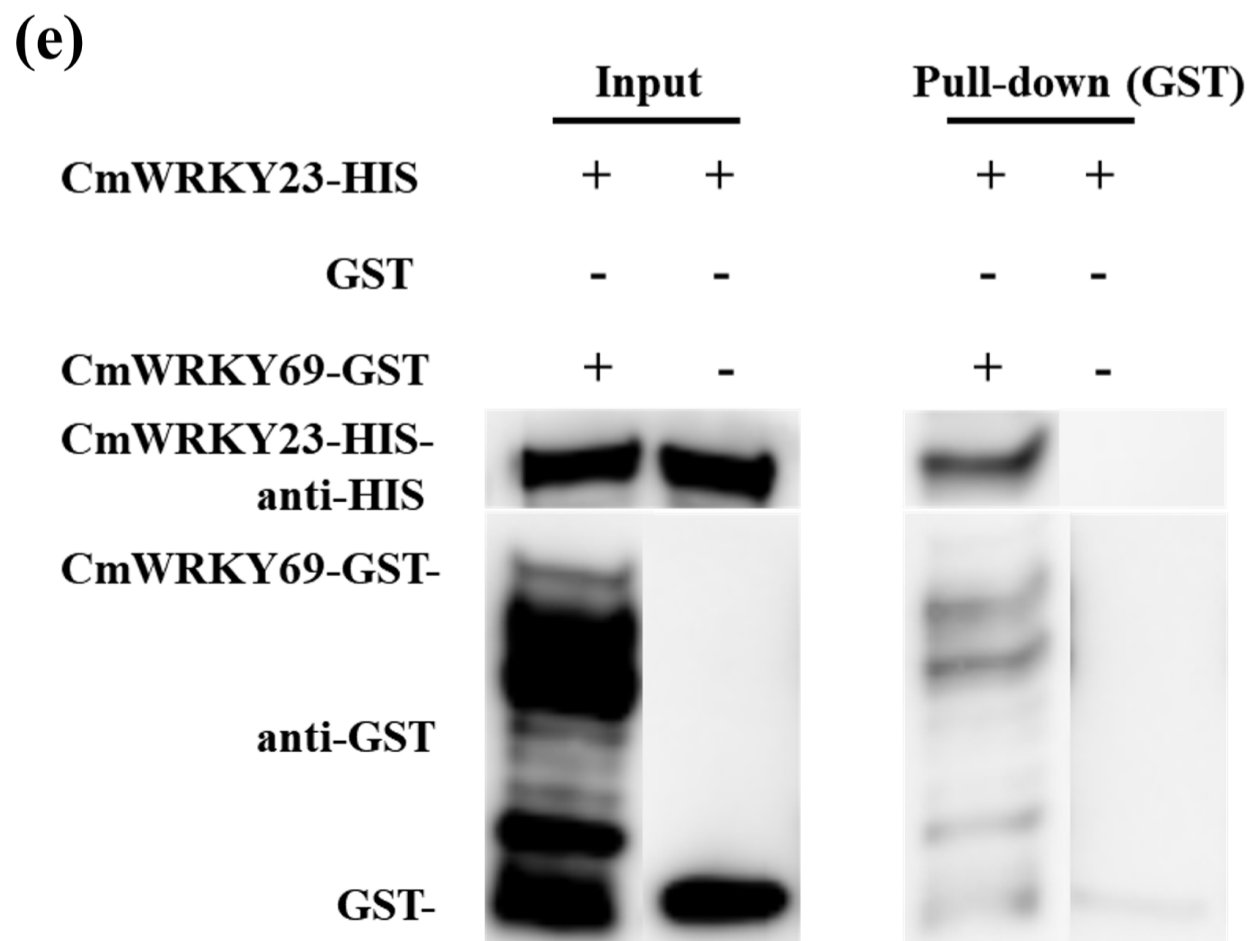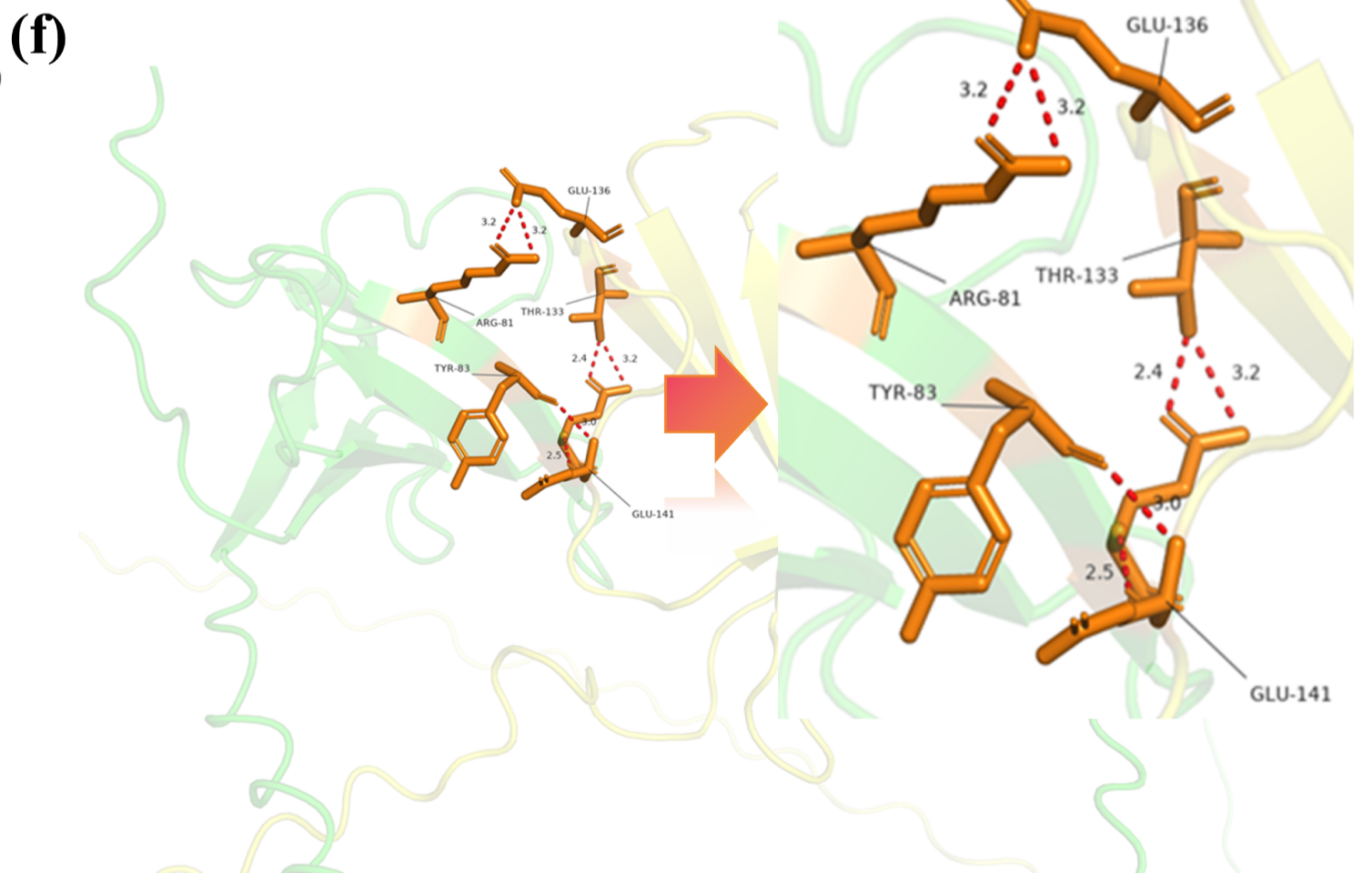

Supplement: Web_Material_uhaf251 [file web_material_uhaf251.zip › Figure 4.pdf]

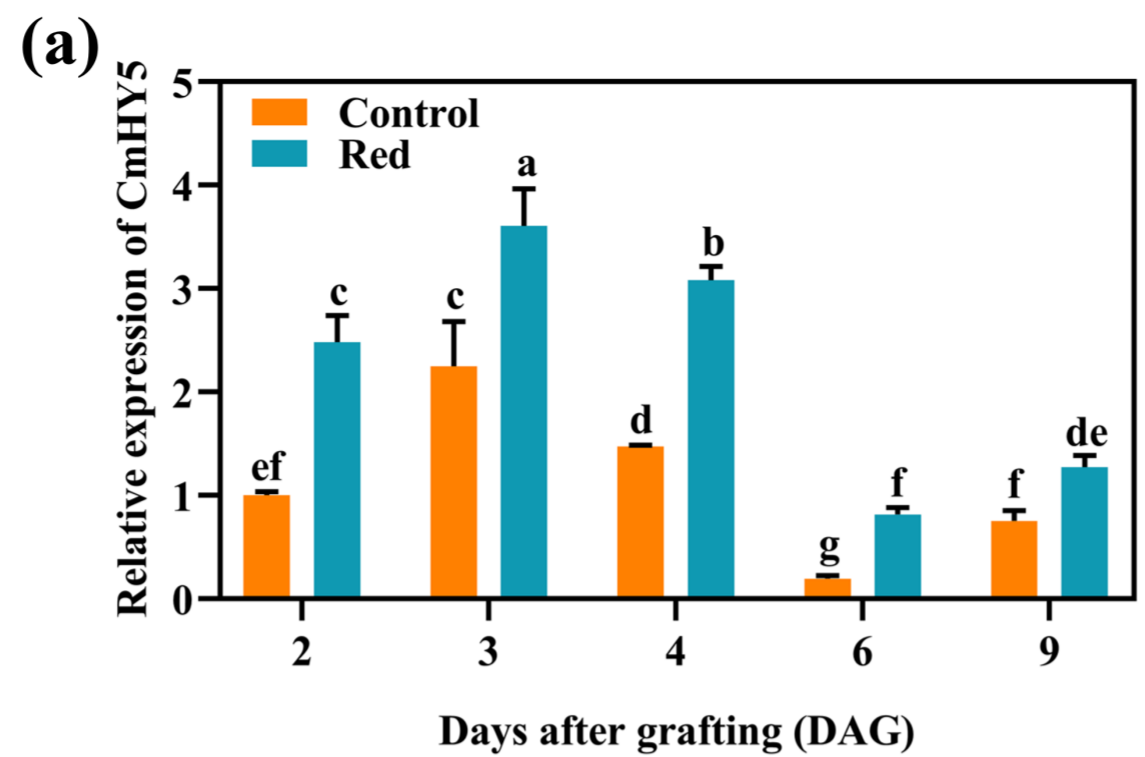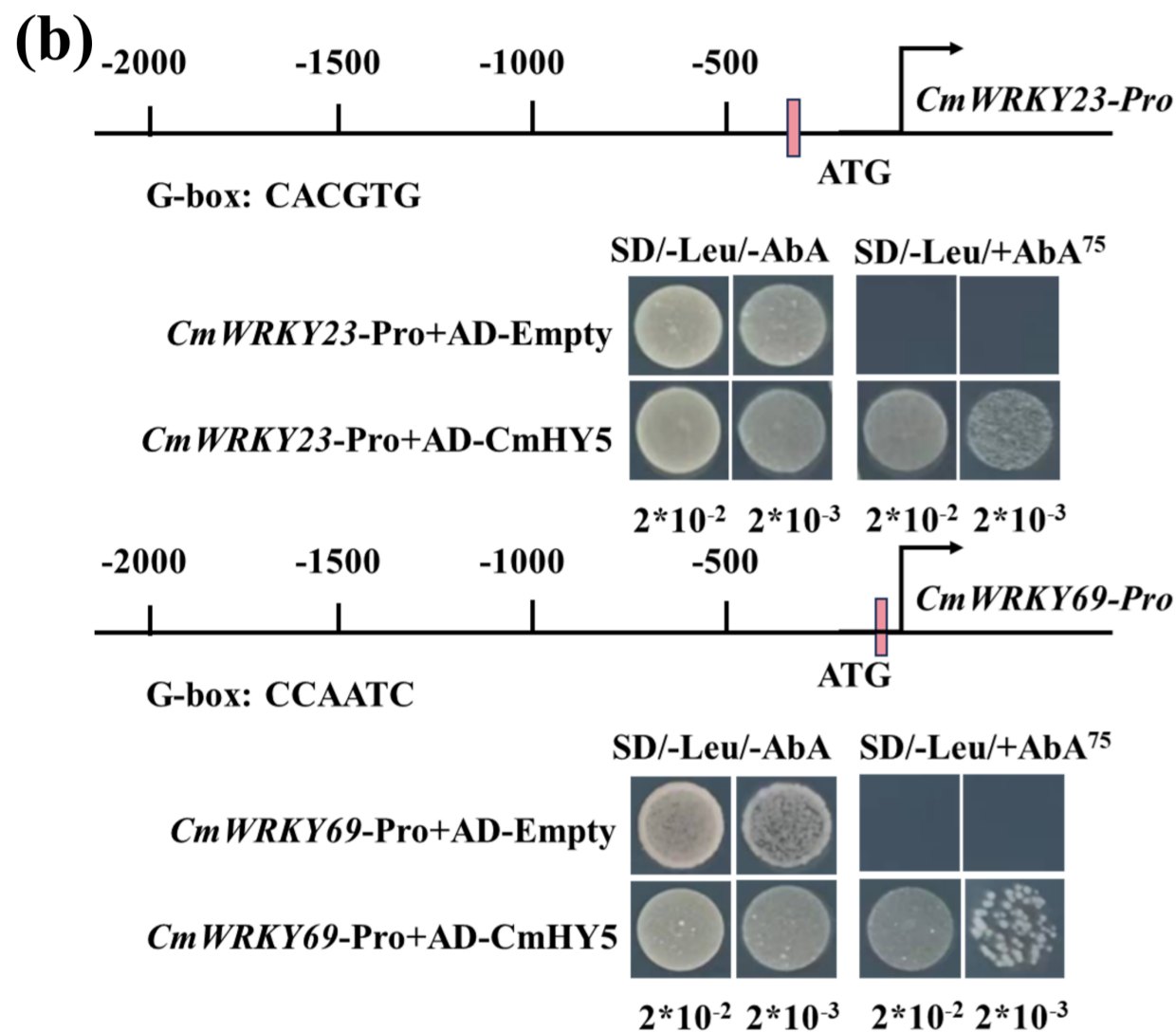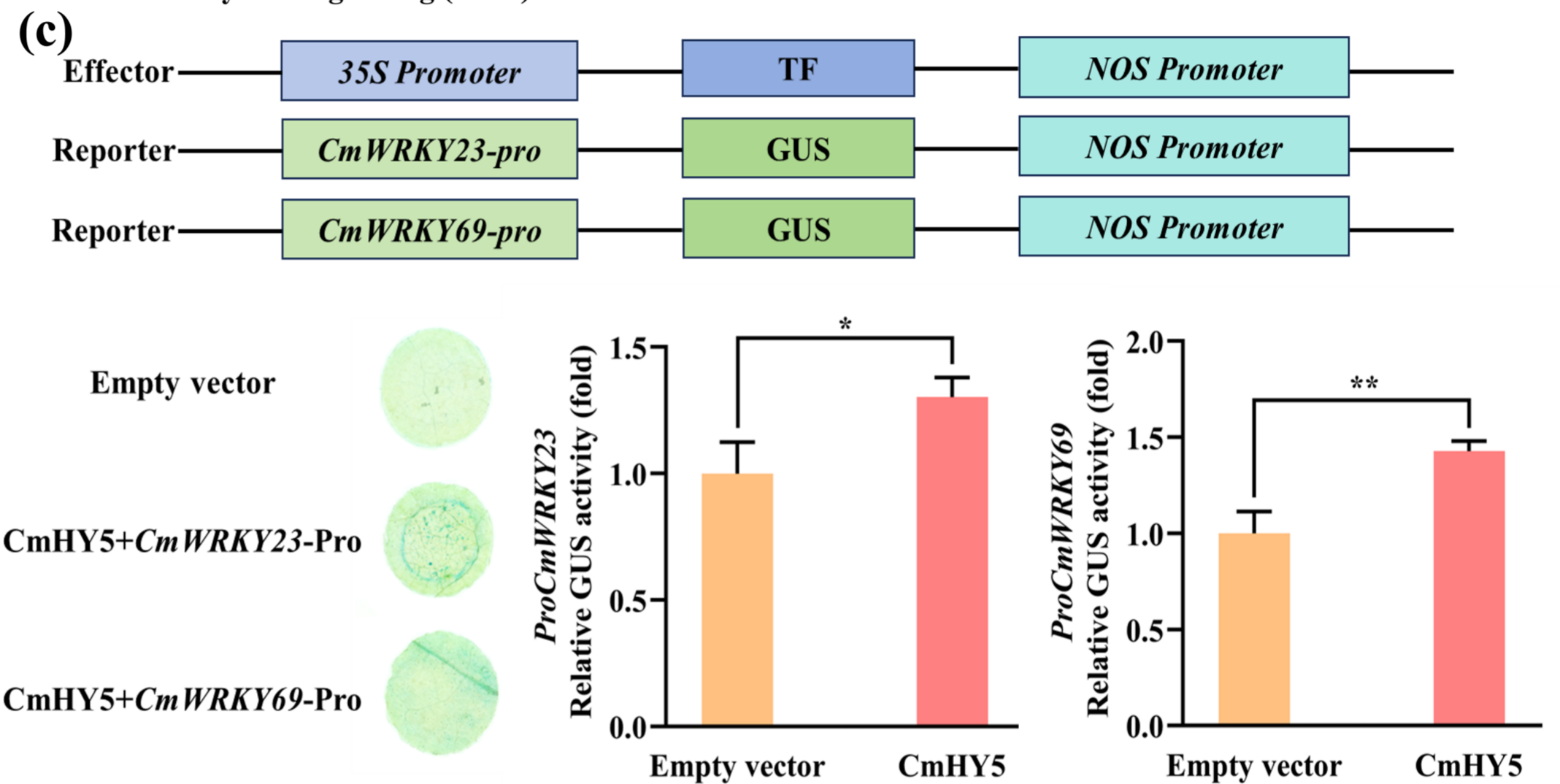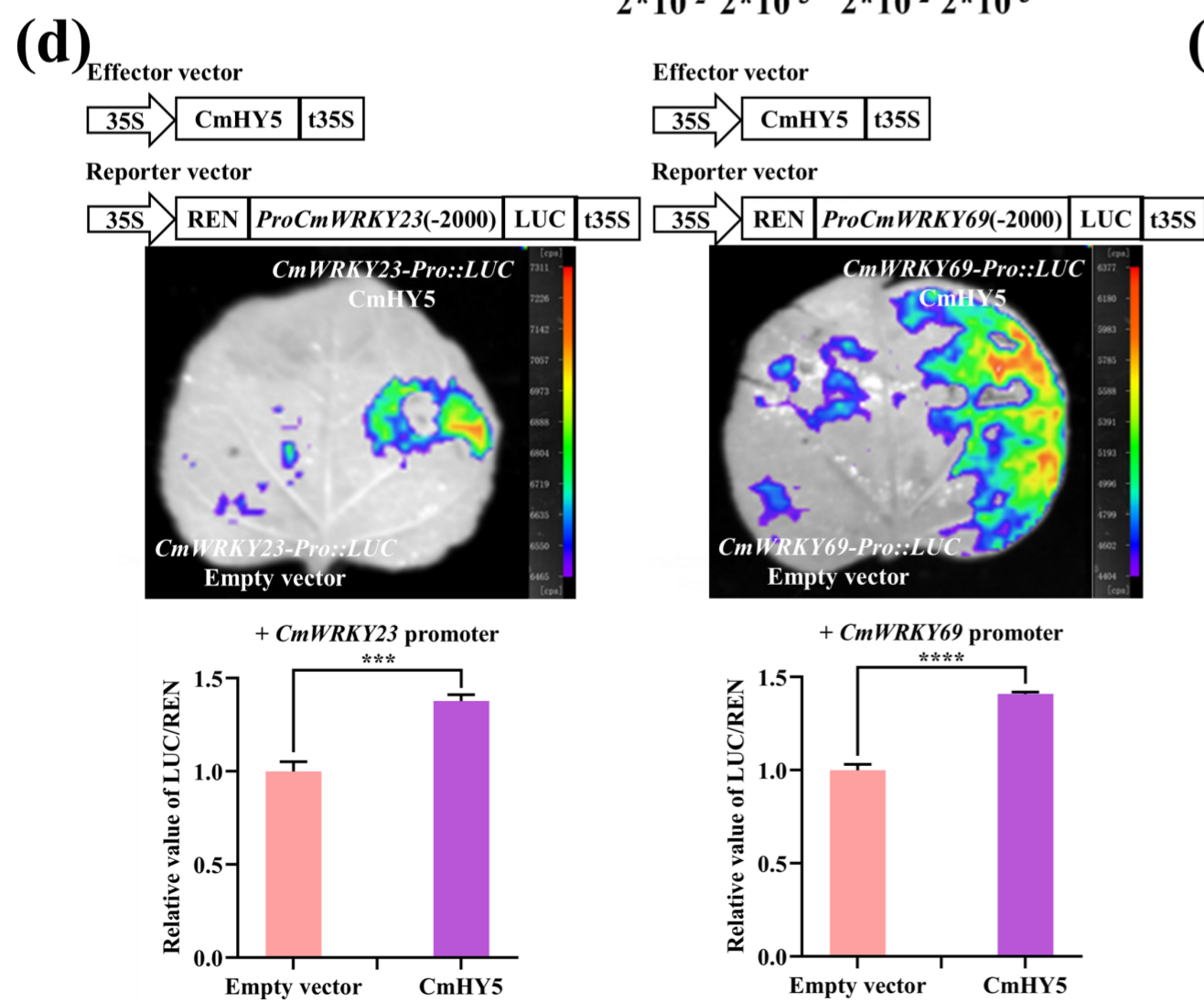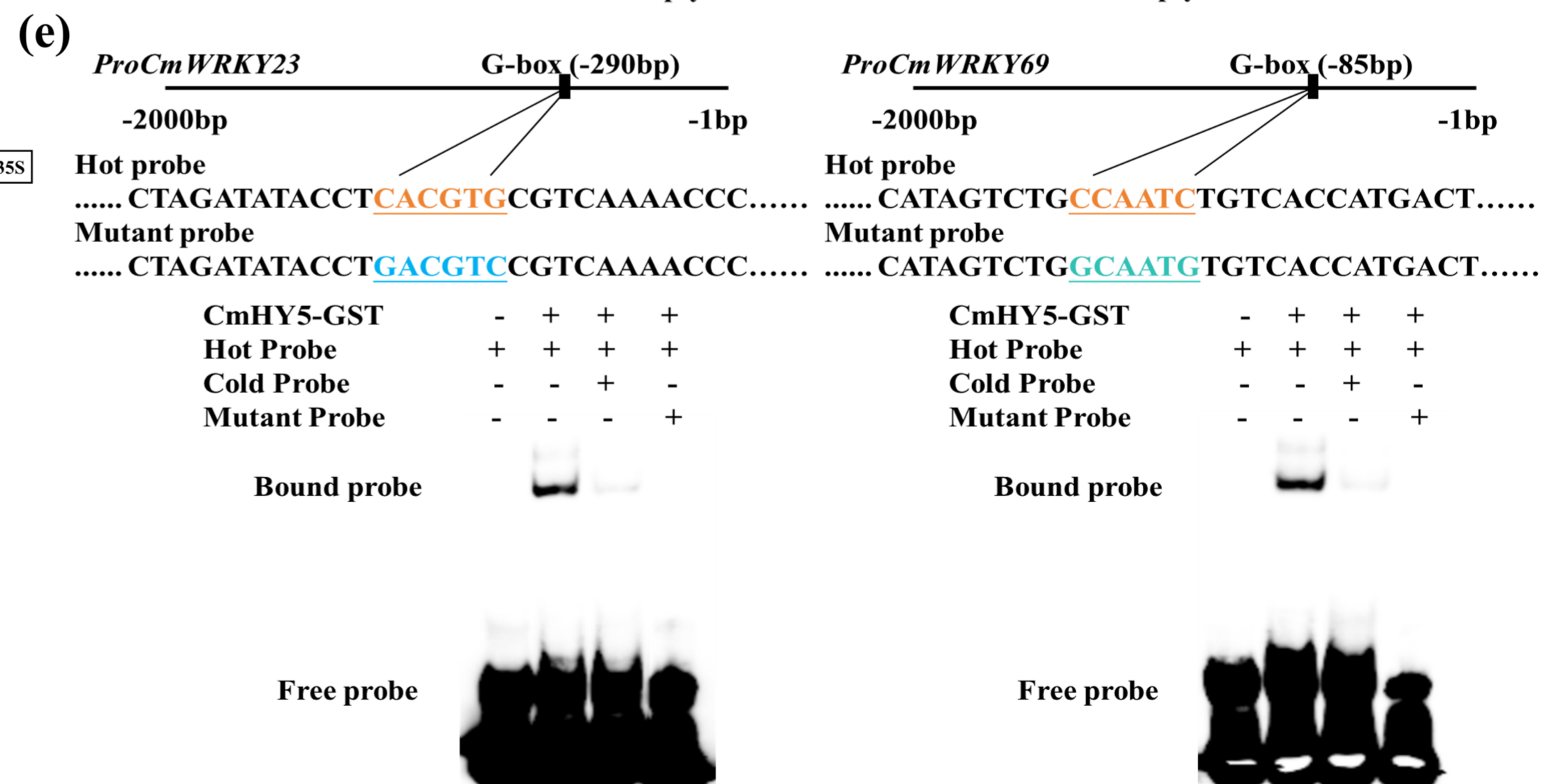

Supplement: Web_Material_uhaf251 [file web_material_uhaf251.zip › Figure 5.pdf]

**(a)**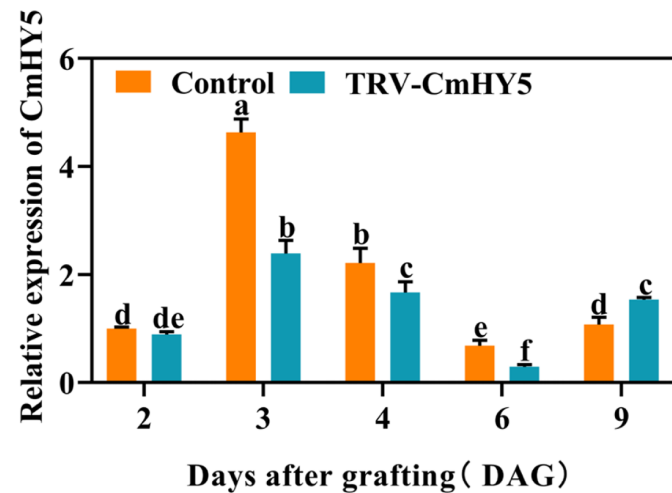**(b)**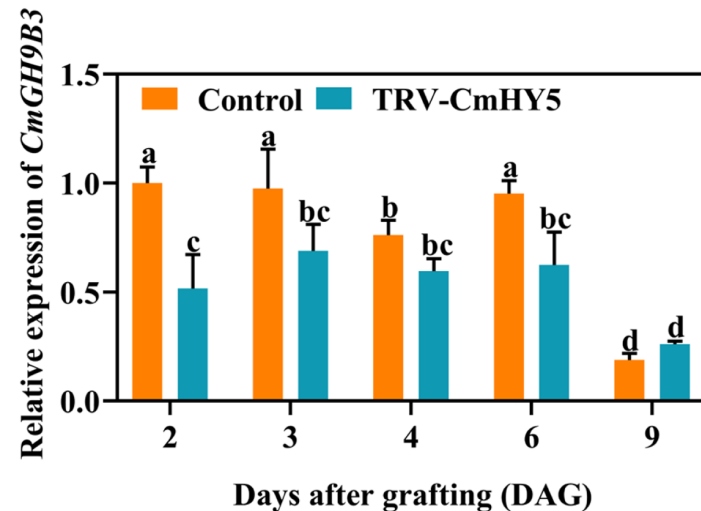**(c)**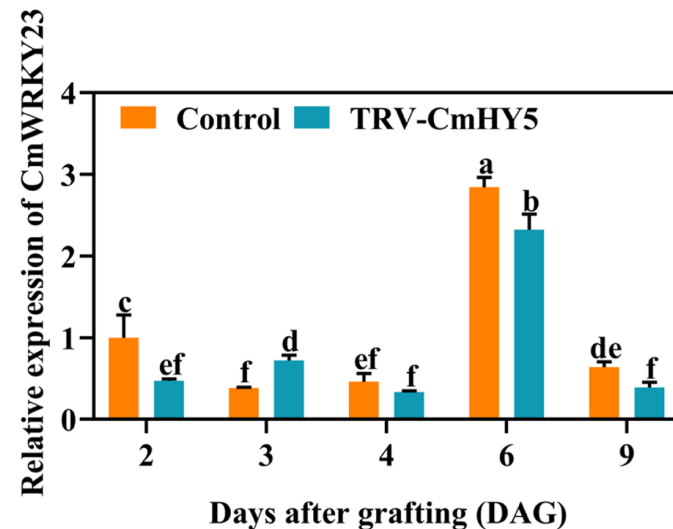**(d)**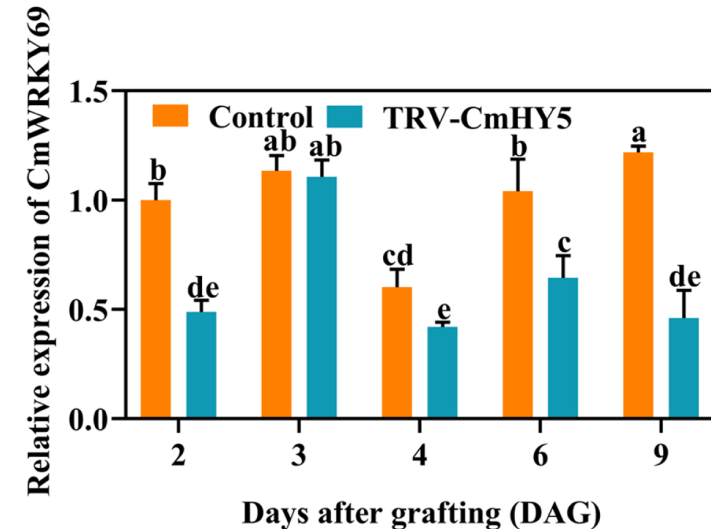**(e)**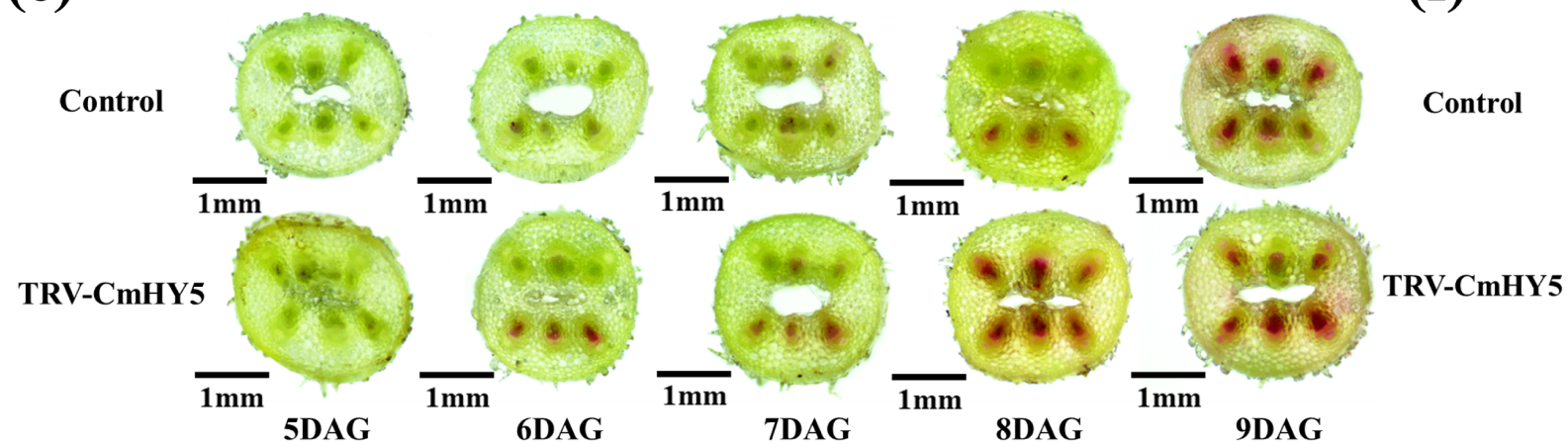**(f)**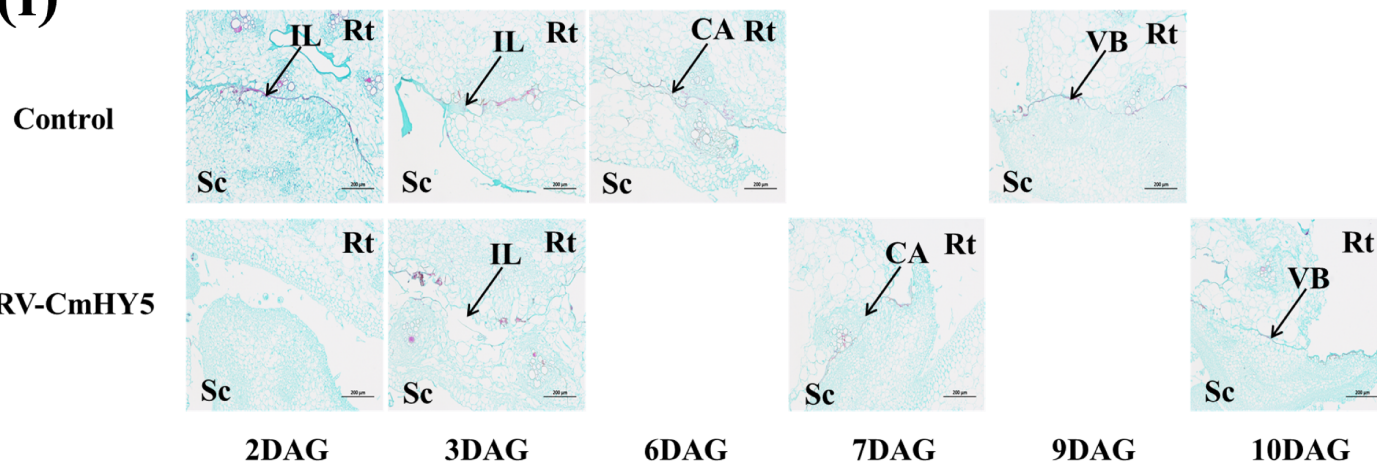

Supplement: Web_Material_uhaf251 [file web_material_uhaf251.zip › Figure 6.pdf]

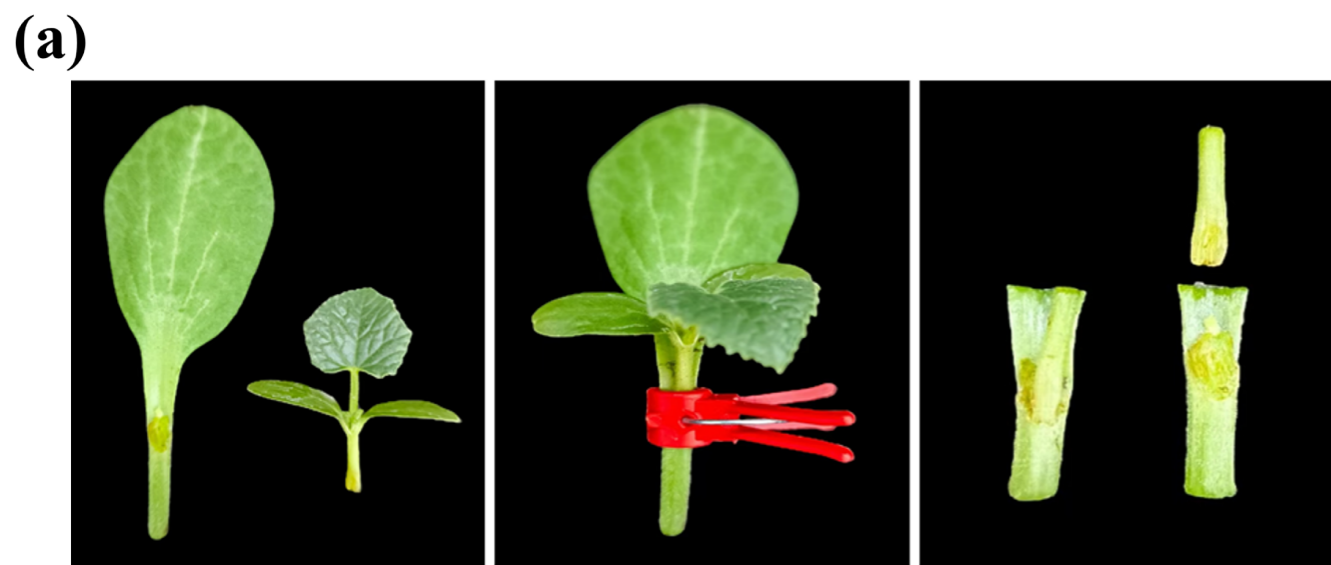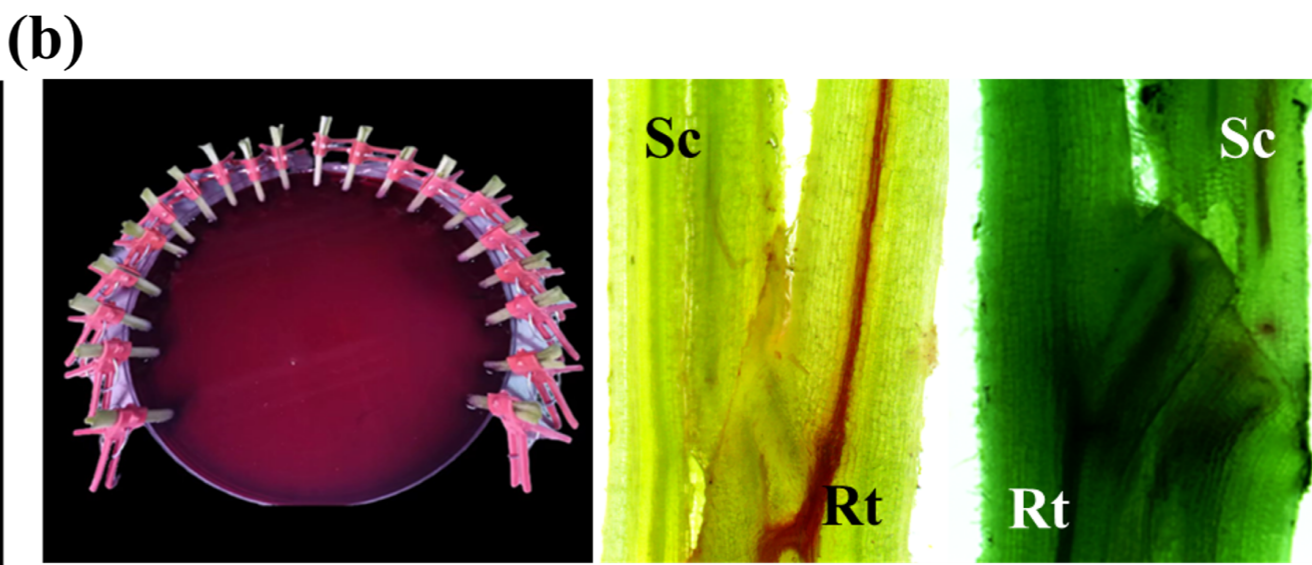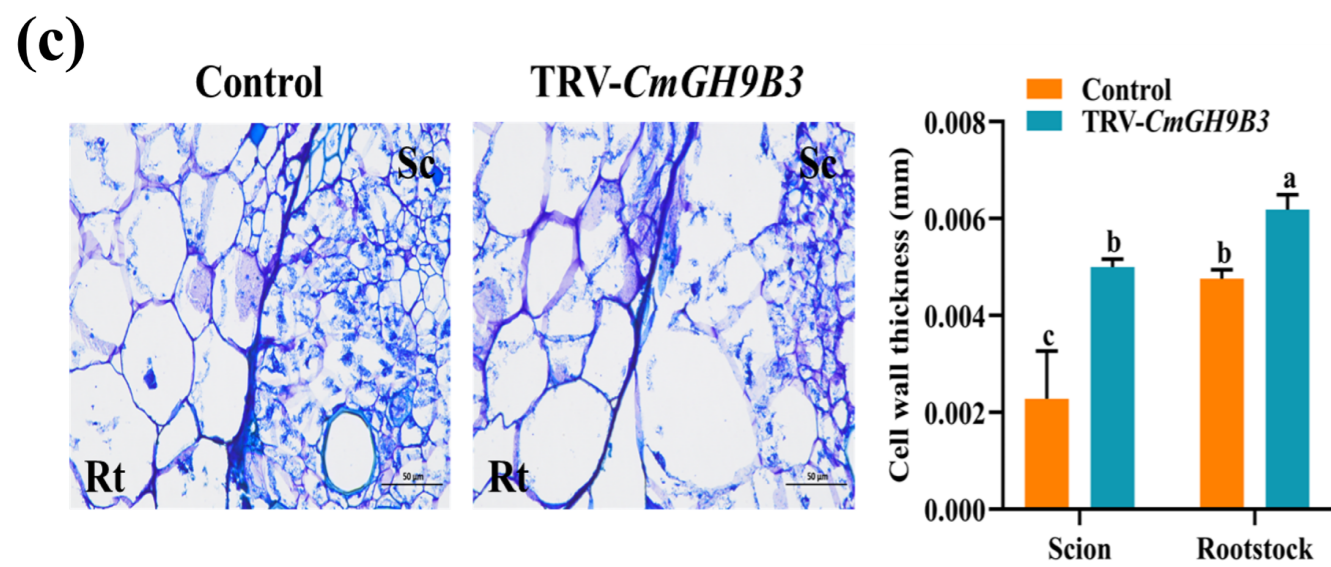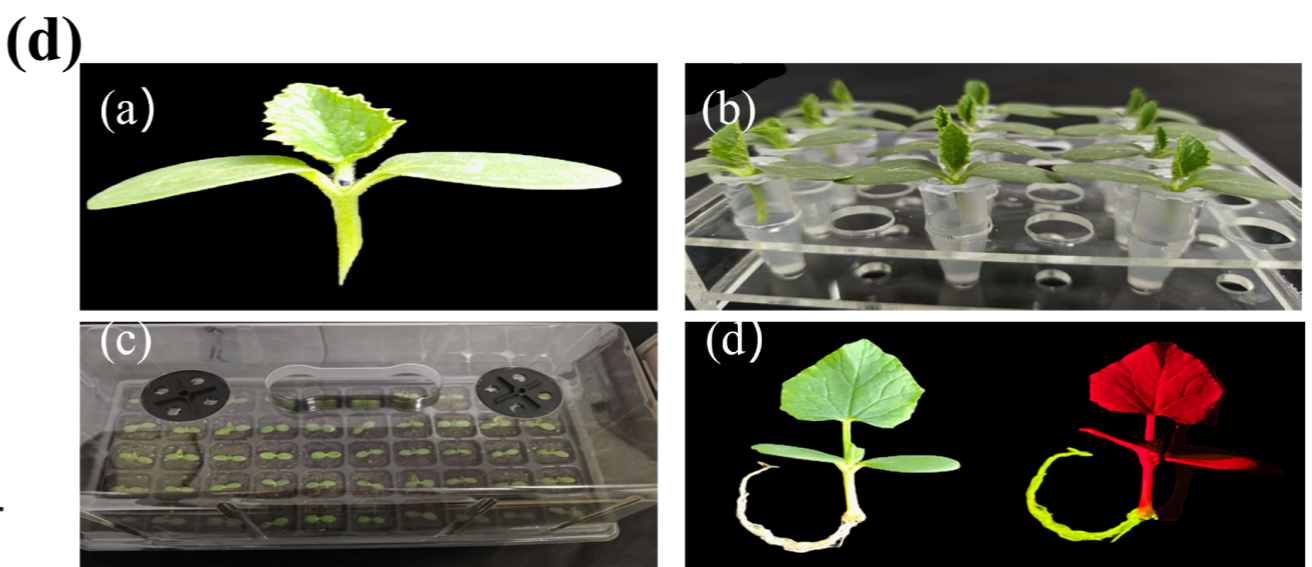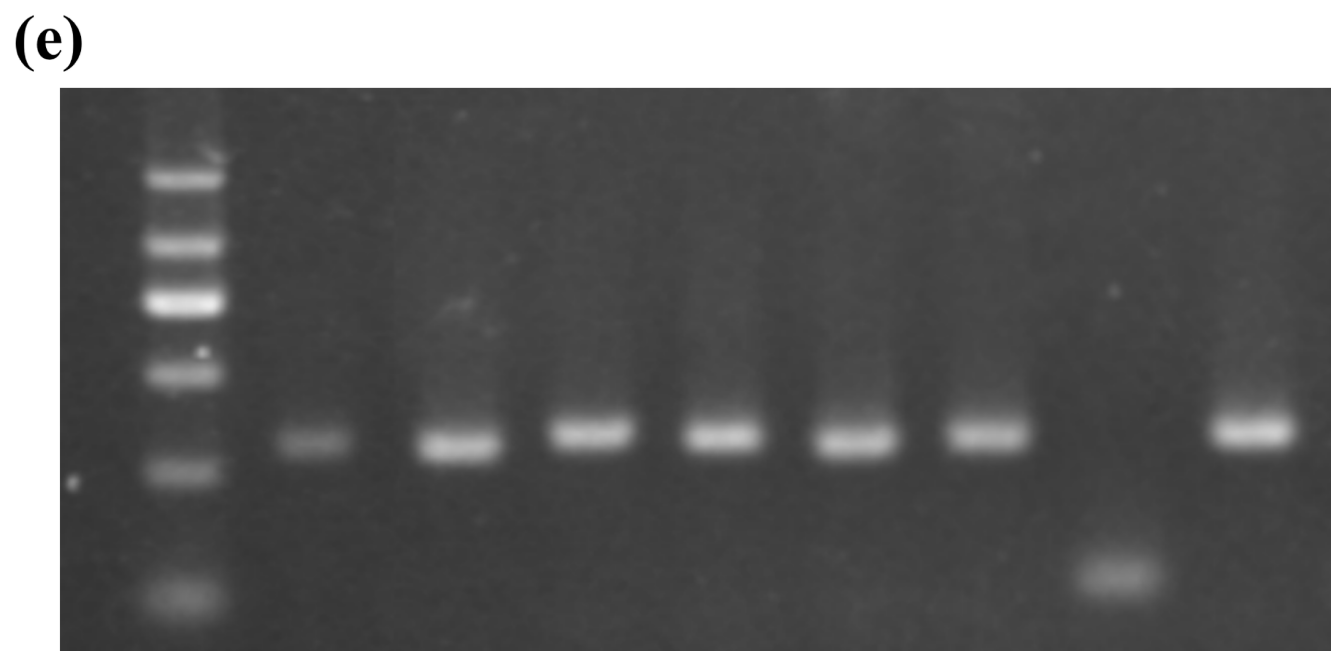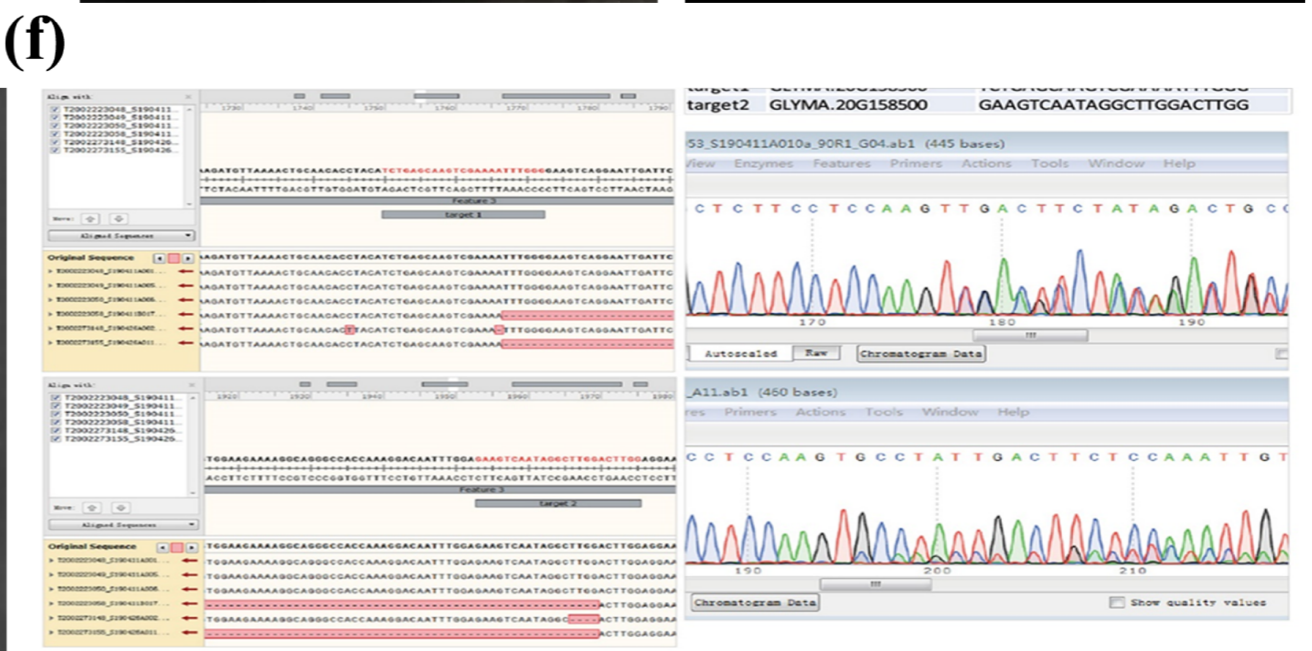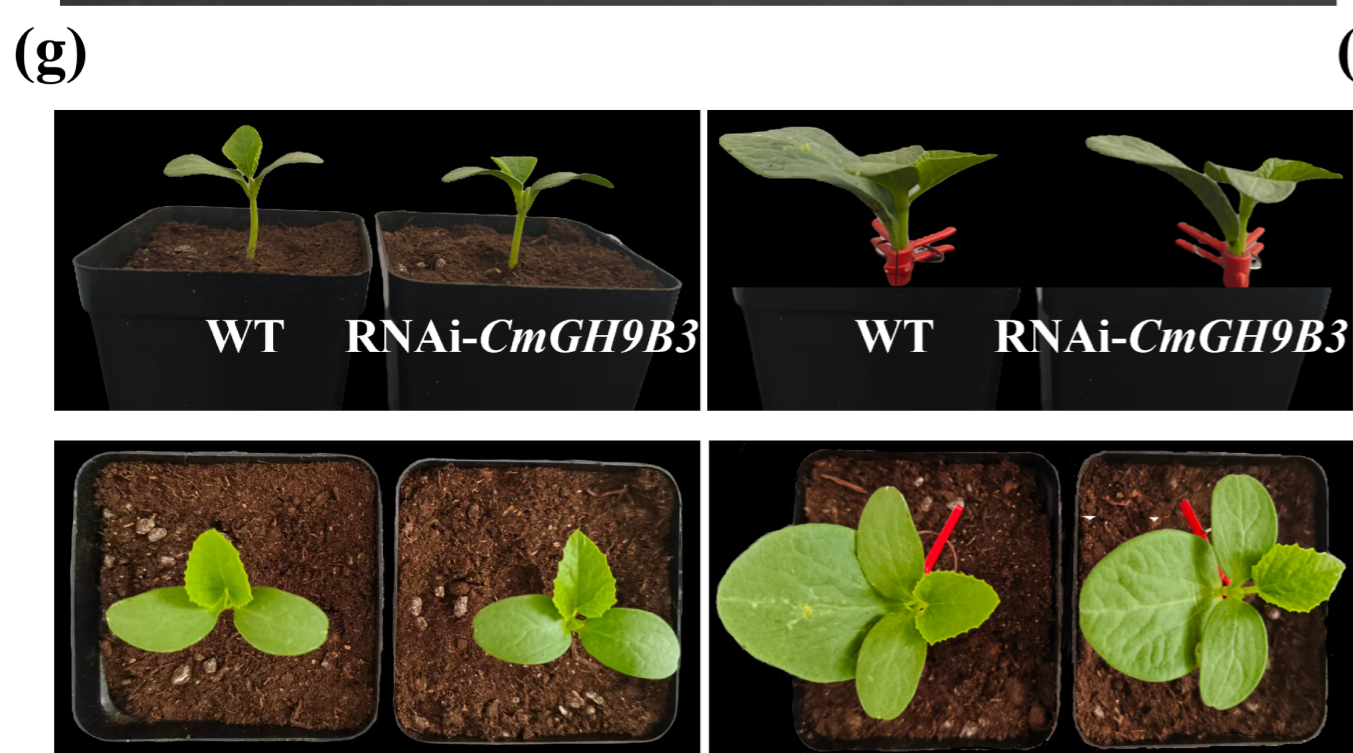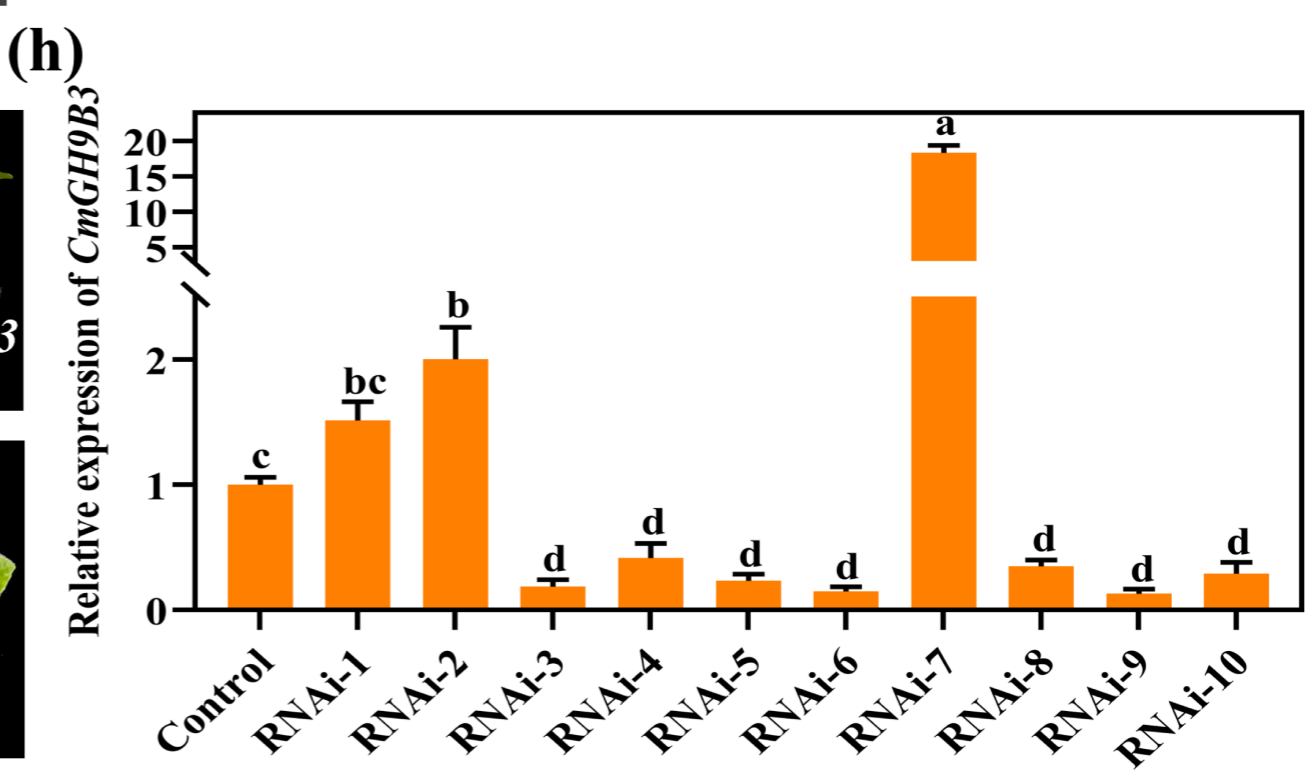

Supplement: Web_Material_uhaf251 [file web_material_uhaf251.zip › Figure S1.pdf]

60min

90min

120min

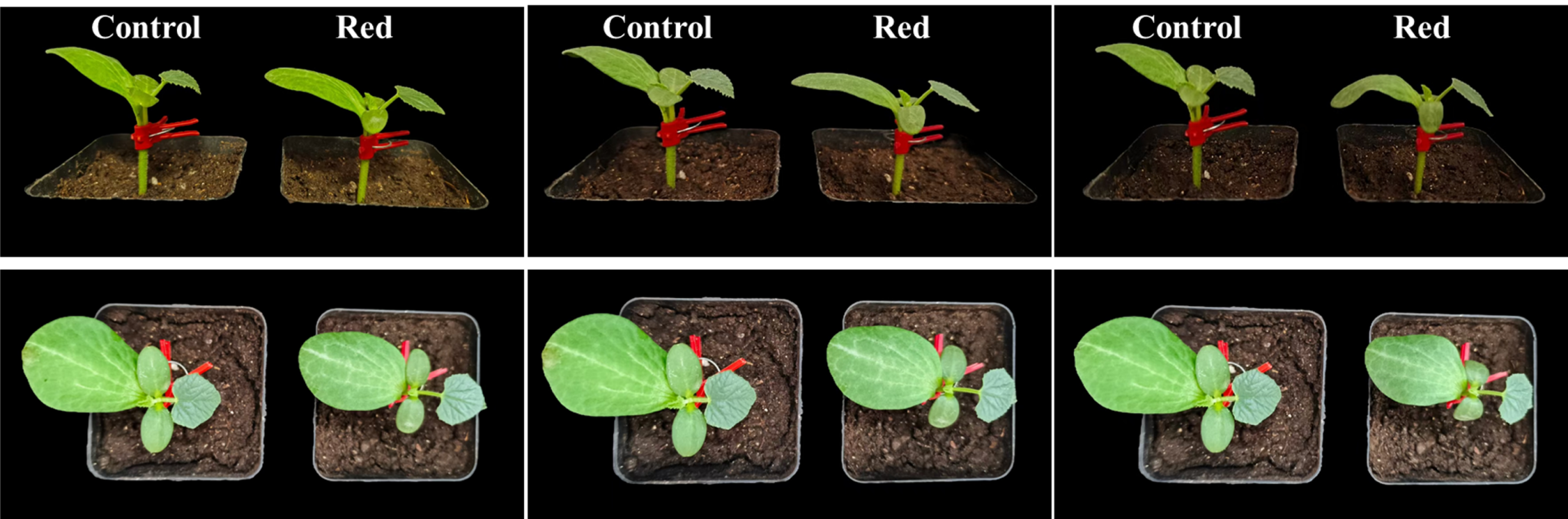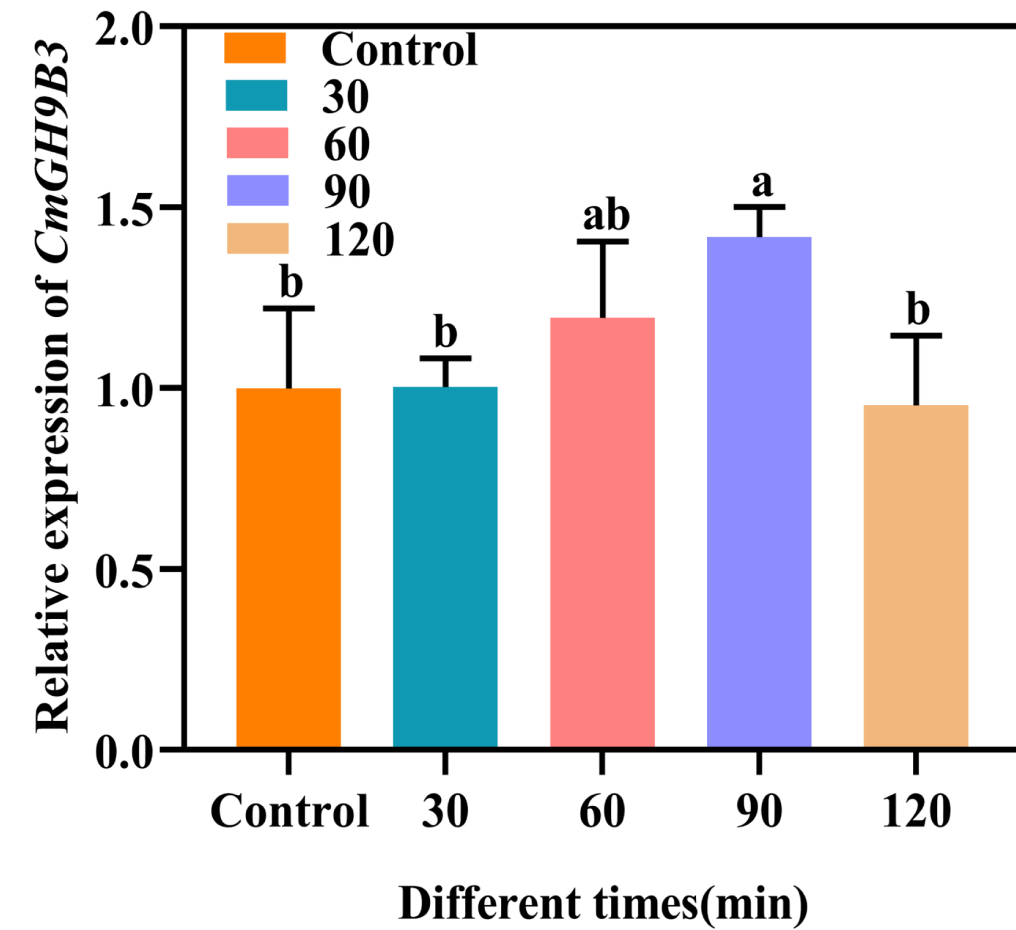

Supplement: Web_Material_uhaf251 [file web_material_uhaf251.zip › Figure S2.pdf]

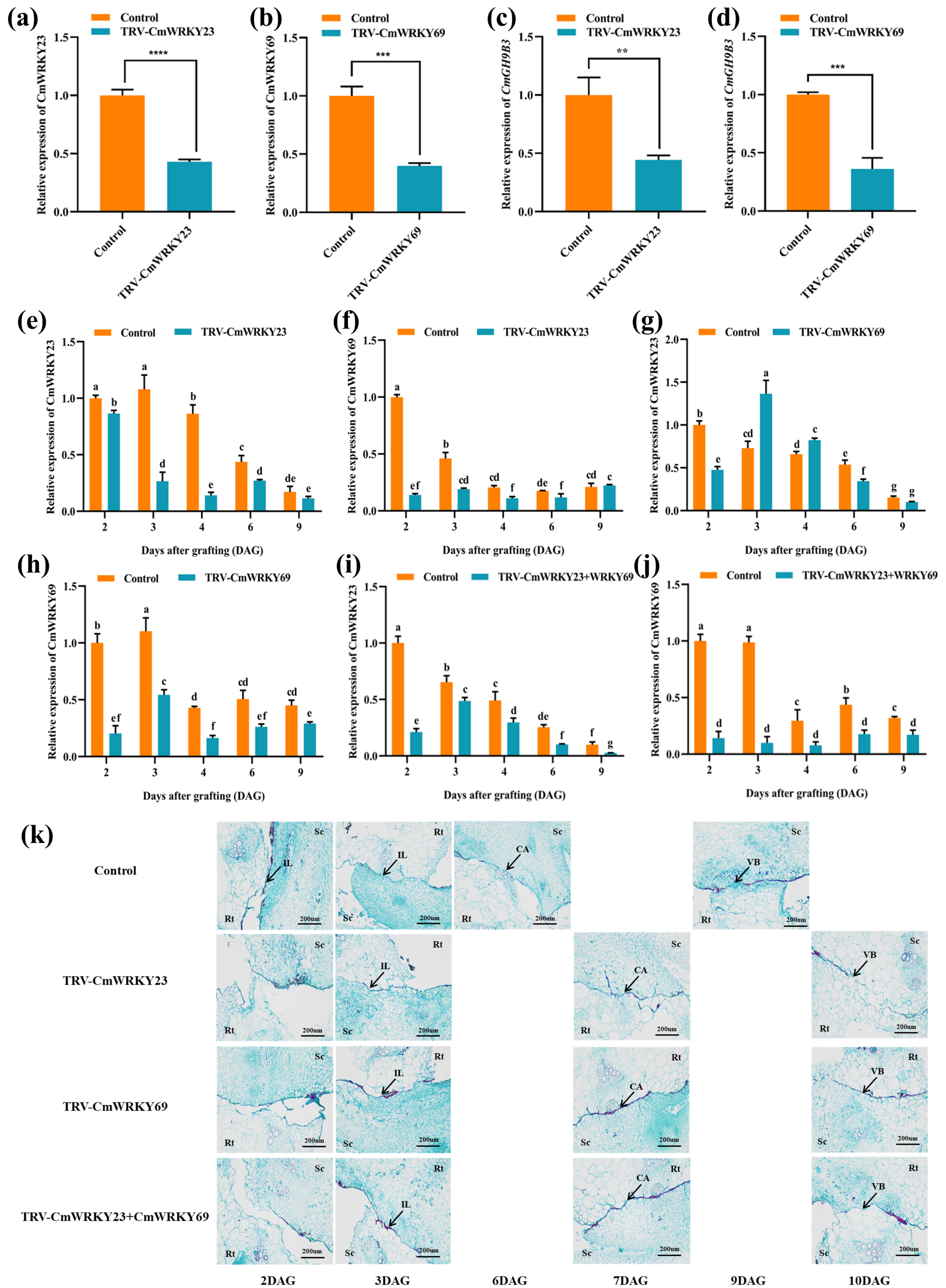

Supplement: Web_Material_uhaf251 [file web_material_uhaf251.zip › Figure S3.pdf]
